# Antimicrobial Activity of *Arthrospira* (Former *Spirulina*) and *Dunaliella* Related to Recognized Antimicrobial Bioactive Compounds

**DOI:** 10.3390/ijms25105548

**Published:** 2024-05-19

**Authors:** Yana Ilieva, Maya Margaritova Zaharieva, Hristo Najdenski, Alexander Dimitrov Kroumov

**Affiliations:** Department of Infectious Microbiology, The Stephan Angeloff Institute of Microbiology, Bulgarian Academy of Sciences, 26 Acad. G. Bonchev Str., 1113 Sofia, Bulgaria; illievayana@gmail.com (Y.I.); zaharieva26@yahoo.com (M.M.Z.); hnajdenski@gmail.com (H.N.)

**Keywords:** antimicrobial activity, extracts, *Arthrospira platensis*, *Dunaliella salina*, cyanobacteria, microalgae, bioactive compounds, two-step process, stress factors

## Abstract

With the increasing rate of the antimicrobial resistance phenomenon, natural products gain our attention as potential drug candidates. Apart from being used as nutraceuticals and for biotechnological purposes, microalgae and phytoplankton have well-recognized antimicrobial compounds and proved anti-infectious potential. In this review, we comprehensively outline the antimicrobial activity of one genus of cyanobacteria (*Arthrospira*, formerly *Spirulina*) and of eukaryotic microalgae (*Dunaliella*). Both, especially *Arthrospira*, are mostly used as nutraceuticals and as a source of antioxidants for health supplements, cancer therapy and cosmetics. Their diverse bioactive compounds provide other bioactivities and potential for various medical applications. Their antibacterial and antifungal activity vary in a broad range and are strain specific. There are strains of *Arthrospira platensis* with very potent activity and minimum inhibitory concentrations (MICs) as low as 2–15 µg/mL against bacterial fish pathogens including *Bacillus* and *Vibrio* spp. *Arthrospira* sp. has demonstrated an inhibition zone (IZ) of 50 mm against *Staphylococcus aureus*. Remarkable is the substantial amount of in vivo studies of *Arthrospira* showing it to be very promising for preventing vibriosis in shrimp and *Helicobacter pylori* infection and for wound healing. The innovative laser irradiation of the chlorophyll it releases can cause photodynamic destruction of bacteria. *Dunaliella salina* has exhibited MIC values lower than 300 µg/mL and an IZ value of 25.4 mm on different bacteria, while *Dunaliella tertiolecta* has demonstrated MIC values of 25 and 50 μg/mL against some *Staphylococcus* spp. These values fulfill the criteria for significant antimicrobial activity and sometimes are comparable or exceed the activity of the control antibiotics. The bioactive compounds which are responsible for that action are fatty acids including PUFAs, polysaccharides, glycosides, peptides, neophytadiene, etc. Cyanobacteria, such as *Arthrospira*, also particularly have antimicrobial flavonoids, terpenes, alkaloids, saponins, quinones and some unique-to-them compounds, such as phycobiliproteins, polyhydroxybutyrate, the peptide microcystin, etc. These metabolites can be optimized by using stress factors in a two-step process of fermentation in closed photobioreactors (PBRs).

## 1. Introduction

Almost every report on the antimicrobial effect of natural products notes that the dangerously rising antimicrobial resistance (AMR) of microbe pathogens necessitates and increases the search for novel sources of antibiotics and other antimicrobials, other than from traditional sources, such as *Streptomyces*.

It is interesting that microalgae and cyanobacteria, such as spirulina, that we associate with food emerge with high antimicrobial activity and contain important antimicrobial molecules [1]. This fact may raise concerns, specifically how Generally Recognized as Safe (GRAS) nutraceuticals could hold anti-infectious therapeutic potential, especially given that the first microalgal antibacterial agent (chlorellin) was discovered in the 1940s [2,3]. Although it has been known for a long time, it has not yet been utilized clinically. However, we must not forget that it is very likely that the antibiotic boom that followed caused chlorellin to be ignored. Pure antimicrobial compounds with low concentrations in GRAS organisms may be isolated and concentrated. The identification of other compounds with direct antimicrobial activity from algae and cyanobacteria is still a relatively young and slow field of research, and new kinds of compounds have been reported in recent years [2,4,5] and research and innovations for commercial application is still in a very early phase [3]. In addition, plants and algae extracts have their specific technological obstructing points for drug development, such as chemical complexity, rediscovery of known compounds, etc., as summarized by Quave (2016) [6].

For those reasons, the fact that terrestrial plant and algal products contribute to just 3% of the nearly 70% natural products and derivatives that are approved by the Food and Drug Administration (FDA) as antibacterial drugs does not accurately reflect their potential [6].

Bactericidal antibiotics and antimicrobials are especially prone to elicit resistance [7], and established antimicrobial agents may cause other serious medical problems, such as destroying normal gut and skin flora and producing gastrointestinal disorders and irritations, dermatitis or serious hypersensitivity problems. Thus, the test of new microbial infection-fighting natural compounds is especially urgent [8].

Natural extracts and compounds also have their well-described benefits for drug development including chirality, the chemical diversity—mentioned also as a drawback—as well as chemical synergy. For instance, complex extracts of *Artemisia annua*. were reported to have antiplasmodial activity that is 6 to 18-fold greater than what was expected based on artemisinin content alone [9,10,11].

In addition, we would like to highlight some other benefits here. Natural products often may not have strong direct bactericidal activity but favor homeostasis towards the host, by enhancing the immunity, etc., thus inhibiting the pathogen [6]. Terrestrial plants and algae used in ethnobotany and traditional medicine have a proven effect and compatibility with the living organism [12,13,14]. Spirulina (present scientific name of the species is *Arthrospira* spp.) has been used as an endurance-booster food from ancient times by Aztecs and natives of Chad, the cyanobacterium *Nostoc* was used by ancient Chinese and *Chlorella* is consumed in the present time [15].

Another benefit of extract synergy is overcoming resistance, and we would like to give the example of Quave (2016) [6] again with artemisinin. It was discovered and crystalized in the early 1970s in China from *A. annua* [16]. Regretfully, it is becoming more and more problematic that resistance to artemisinin monotherapy appeared in a short period of time and is globally spreading [17]. However, *A. annua* is a traditional Chinese medicinal plant named Qinghao, and it is a therapy known to have been in use for millennia and has not yielded resistance [16]. Whole plant therapy was even effective at overcoming artemisinin resistance in an animal model [9,10,11]. These studies supported the idea that the synergetic action of multiple compounds in this species can overcome resistance noted in monotherapy, apart from other factors, such as the widespread use of the drug due to global distribution. This idea might apply in the future to the creation of novel antimicrobial formulations from natural products intended to prevent the development of resistance [6].

Regarding the taxonomy of microalgae, there is not yet a full consensus among academia whether cyanobacteria such as *Arthrospira* and *Nostoc* are (micro)algae, as they would be nearly the only group of the prokaryotic Bacteria domain in this category. Many credible scientific sources still include them, as the tradition is, since (micro)algae is an informal polyphyletic term, meaning it does not form a natural group that has descended from a common ancestor. Its eukaryotic members are species from multiple distinct clades and both from the kingdom Plantae sensu lato and not [18,19,20,21]. Cyanobacteria and eukaryotic microalgae share many traits in terms of physiology, ecology and, particularly, biotechnological applications [22]. We will collectively refer to them as phytoplankton.

The medicinal potential of microalgae in brief includes their ability to synthesize anti-inflammatory antioxidants, such as carotenoids, vitamin E [23] and polyunsaturated fatty acids (PUFAs). PUFAs are vital for human metabolism and cardiovascular health, two of them with longer chains even being prescription drugs for lowering triglyceride levels [1,24,25,26,27]. In fact, marine microalgae are the main primary producers of those longer chain ω-3 PUFAs [28]. As for the only approved drug derived from a unique to microalgae (cyanobacteria) compound, it is the anticancer drug dolastatin [29].

Microalgae also produce sterols with pharmaceutical application and cytostatic phycotoxins [23] and also have immunomodulatory, anti-obesity and other effects [1]. Microalgae are reported to be one of the most important producers of antimicrobial molecules, including PUFAs and other fatty acids, proteins, vitamins, pigments, etc. [1].

Spirulina is the consumed biomass of filamentous nutraceutical cyanobacteria and may consist of *Arthrospira platensis* Gomont (synonym *Arthrospira fusiformis* or formerly *Spirulina platensis*) or *Arthrospira maxima*. It is approved by the FDA as GRAS and sold as a food supplement, largely because of the 65% protein in the dry mass. *A. platensis* inhabits tropical lakes with alkaline waters (pH 11) and a high concentration of salt and bicarbonates. While these circumstances hinder the development of other microorganisms, they permit the cultivation of spirulina in open reactors. *A. platensis* and *A. maxima* inhabit highly alkaline lakes in Africa and Mexico, while other *Arthrospira* spp., besides brackish water, can be practically found everywhere—seawater, freshwater, soil, marshes and thermal springs [30,31]. The protein of spirulina contains all the essential amino acids, and *A. platensis* contains 10% *w*/*w* carbohydrates and 7% *w*/*w* lipids, of which 1.5–2% are PUFAs [32]. *Arthrospira* is a substantial source for complementary and alternative medicine and has a significant potential in conventional medicine because of its proven in vitro and in vivo bioactivities [30,31,33]. It has immunostimulant, antioxidant, anti-inflammatory, antimicrobial, antineoplastic and a broad spectrum of antiviral activity [30]. Spirulina is effective against certain allergies and rhinitis and has antidiabetic, anti-obesity and metalloprotective hepatoprotective properties. It is also reported to influence neurodegenerative disorders, anemia, cardiovascular diseases, hyperglycemia and hyperlipidemia. These activities are attributed, individually or synergistically, to *Arthrospira*’s bioactive compounds. In fact, cyanobacteria such as *Arthrospira*, *Anabaena*, *Nostoc* and *Oscillatoria* are claimed to synthesize a great variety of secondary metabolites comparable to the metabolite diversity of actinomycetes [34]. *Arthrospira* is rich in fatty acids, including essential omega-3 or omega-6 PUFAs, β-carotene, α-tocopherol (vitamin E), phycocyanin, phenol compounds, caffeic and chlorogenic acid, dietary minerals and vitamins and a sulphated polysaccharide with antiviral property (calcium spirulan) [30,31,33].

*Dunaliella salina* (Dunal) Teodoresco is a halophile green microalga. It is especially found in hypersaline environments, such as salt lakes and salt evaporation ponds, where it contributes to their pink color. It has high concentrations of glycerol to counter the salinity by osmotic pressure and high concentrations of β-carotene. *Dunaliella* has two flagella and a single cup-shaped chloroplast containing the β-carotene, which makes it appear orange-red. It is nutrient rich, hence used for dietary supplements [35]. *Dunaliella* spp. can also be found in marine and freshwater habitats [36].

Although products from the two genera have been so far developed only into food supplements but not into approved drugs, we must not underestimate the former. Dietary supplements can also be part of clinical therapeutic schemes for chronic diseases, e.g., kidney stones.

**Figure 1:**
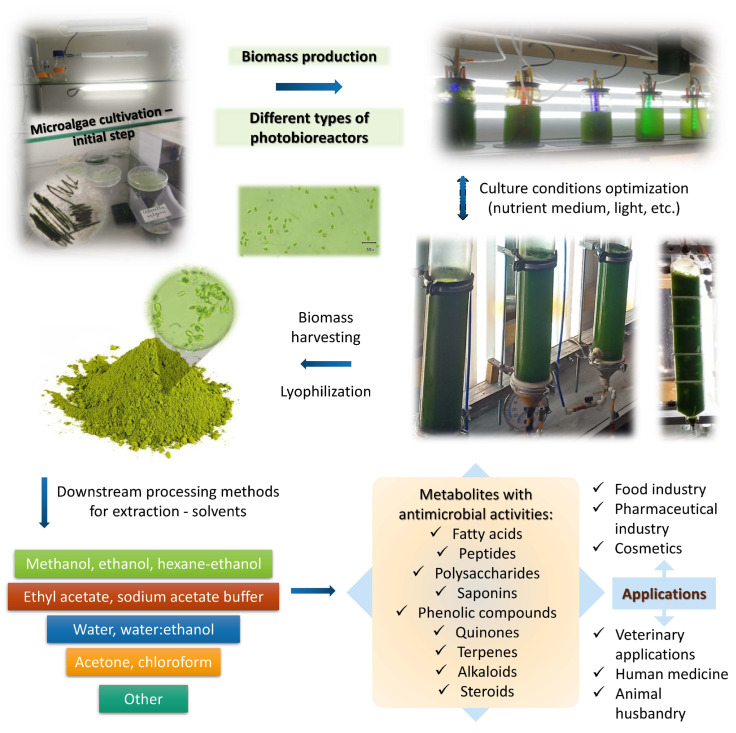
Production and isolation of antimicrobial metabolites from microalgae and cyanobacteria and possible applications—flow diagram of the process.

The topic of bioactive and nutraceutical metabolites is also relevant to the circumstances under which the organisms, in our case, microalgae or phytoplankton, of interest optimally synthesize secondary metabolites. Since uncontaminated microalgal biomass can be obtained from bioreactors and not from the field, authors have to highlight the main strategy of boosted production during controlled fermentation conditions. Theory in the field is well established, and the two-step process of maximization of secondary metabolites is well studied and proven. The first step consists of maximum growth rate by optimal conditions, and the second step applies stress factors, which induce and increase some secondary metabolites. For example, carotenoids, fatty acids and sulphated polysaccharides have been reported to increase after various chemical triggers, such as low oxygen and salt and nutrient starvation in *A. platensis*, *D. salina*, *Chlorella zofingiensis,* etc. [37]. All this is related to the trend of cultivating *Arthrospira* in bioreactors instead of open ponds, which provides an increase in biomass due to the lack of water evaporation and a higher quality of the product (open ponds still have the benefit that control of temperature and light is not necessary) [30]. Figure 1 presents a schematic diagram of production and isolation of antimicrobial metabolites from phytoplankton. More details in regard to the stress factors can be found in the corresponding section.

Reviews covering some of the reports on the antimicrobial activity of *Arthrospira* and *Dunaliella*, in addition to other phytoplankton species, are those of Falaise et al., 2016 [38], and Senhorinho et al., 2015 [39]. This review aims to comprehensively describe and analyze the reported in vitro and in vivo antimicrobial activity (antibacterial and antifungal) of the genera *Arthrospira* and *Dunaliella* as well as to provide some focus on their bioactive compounds and the strategies for their enhancement in bioreactors.

## 2. *Arthrospira* and *Dunaliella* Are among the Most Commercially and Industrially Used Cyanobacteria and Microalgae in Biotechnology

*Arthrospira* and *Dunaliella* are among the most exploited phytoplankton species in biotechnology. As the reviews of Mobin et al., 2017 [36], and Bhalamurugan et al., 2018 [40], outline, the reason is that they are rich sources of high-value compounds. The large amount of obtained biomass that is already used for biotechnology purposes would favor the production/extraction of antimicrobial high-value products. Overall, this strategy would result in diminishing the cost of the microalgal technology.

Over the last 20 years, four major phytoplankton species—*Arthrospira*, *Chlorella vulgaris*, *D. salina* and *Haematococcus pluvialis*—have been utilized in biotechnology [36,41]. The most commonly exploited microalgae and cyanobacteria for the production of bioactive compounds with pharmaceutical purposes include *Arthrospira*, *Chlorella*, *Dunaliella*, *Haematococcus* and *Nostoc* [40].

To date, *Arthrospira* (together with *Chlorella*) has been the most commonly sold phytoplankton species for food because of its robust growth. In fact, only a few from the approximate 40,000 species of algae and cyanobacteria have been used by the food industry, and *Dunaliella* spp., as well as *A. maxima* and *A. platensis* (still frequently and popularly named spirulina), are among them [42]. They are used as a supplement to enhance the nutritional and health benefits of breads, candies, ice cream and other common foods, pastry and sweets. They are directly manufactured into health products like tablets and capsules and also used as food coloring [1]. *Arthrospira* spp. are very valuable, as they are rich in proteins, carbohydrates and vitamins. Their proteins and vitamins, especially B12, are used for antioxidants and immune enhancers. The genus is rich also in essential amino acids, minerals, essential PUFAs, etc. Due to this, it holds second place in (health) beverage production. Proteins of the nutrient-rich *D. salina* are also used in the baking industry [36,43].

However, *Arthrospira* and chlorophytes, such as *Dunaliella* and *Chlorella*, are not suitable as a single-species diet, as they are not rich sources of eicosapentaenoic acid (EPA) and docosahexaenoic acid (DHA) [25,26,44]. Microalgae may be the main source of the physiologically important ω-3 PUFAs EPA and DHA in fish oil; however, this role is attributed mainly to dinoflagellates [45].

According to a report by The Food and Agriculture Organization, the global production of *Arthrospira* sp. was 56,000 t in 2019 [41]. The increase in global production of species, such as *Arthrospira* (3000 t dry weight/y), *D. salina* (1200 t dry weight/y) and, e.g., *Chlorella* (2000 t dry weight/y), indicates a positive trend of human consumption, which will likely rise even more in the years to come. Microalgae and cyanobacteria production has a compound annual growth rate of 5.32% [40].

*Dunaliella* and *Arthrospira* (together with *Chlorella* and *Scenedesmus*) are also among the commonly used species of microalgae for animal and aquaculture feed, especially because they can grow in highly saline and alkaline medium [43].

Phycobiliproteins are photosynthetic pigment–protein complexes found within cyanobacteria, in addition to chlorophyll. Their major sources are *A. platensis*, other *Arthrospira* spp. and *Amphanizomenon floaaquae*. Examples of these proteins include phycocyanin and phycoerythrin. In fact, the biomass from *A. platensis* and *Arthrospira* spp. is claimed to be used mainly for the extraction of phycocyanin. All phycobiliproteins are used in the pharmaceutical industry, as they are antioxidant, anti-inflammatory, neuroprotective and hepatoprotective agents and are utilized in photodynamic therapies of various solid cancers and leukemias. Next, they are used in clinical laboratories and immunology research due to their effective molecular absorption, high fluorescence ability for marker production and photostability. Last, but not least, they are used commercially as natural food dyes and in cosmetics in perfumes and eye makeup [36,46,47,48].

*Dunaliella salina* is the best source of β-carotene (up to 14% of dry biomass), followed by *Scenedesmus almeriensis*, and the sole source of naturally derived β-carotene. This carotenoid is an antioxidant and a vitamin A precursor. Another commonly used microalgae for production of β-carotene is *Dunaliella bardawil* (also a GRAS algae). The amount of β-carotene it produces is 1.65 pg/cell with an approximate market value of 0.6 US$ per 1000 mg of β-carotene [49]. *D. salina* is responsible for most of the primary production in hypersaline environments worldwide and also a source of glycerol [35].

The carotenoids lutein, zeaxanthin and canthaxanthin have pharmaceutical value mainly as antioxidants for health supplements and in the food industry as natural colorants (interestingly, for, e.g., chicken skin). *Dunaliella salina* is among the most utilized microalgal producers together with some *Chlorella* and other species. *Arthrospira* is also rich in zeaxanthin [50].

Other pharmaceutical benefits of the two genera include sulphated polysaccharides of *Arthrospira*, which are widely used as an antiviral agent and tablets, being marketed since 1975 in Japan [36]. Humans have eaten *Arthrospira* to lower arterial pressure, induce the growth of intestinal *Lactobacillus* and reduce hyperlipidemia [48].

*Dunaliella salina*’s oxidized carotinoids, or xanthophylls, have anticancer properties. Additionally, that microalga has potential to create broncholytic, analgesic and antihypertensive medications [36].

*Dunaliella* and *Arthrospira*, in addition to *Haematococcus* and *Chlorella*, are commonly found in a variety of cosmetics. Lotions and creams for the face and body are replenished with these microalgae extracts. They are also an ingredient in sun protection creams, hair masks and shampoos. They also help in the regeneration of fibers and stop wrinkles from developing on the surface of the skin. *Arthrospira* extract enhanced with proteins slows down the aging process of the skin [48,51,52].

*A. platensis*, as well as *Chlorella* and *Scenedesmus* spp., and are among the most commonly used phytoplankton species for bioethanol production [40]. Other industrial uses of the two genera include wastewater treatment. Many microalgal species may find the produced water from the oil and gas industries toxic, but *Dunaliella* spp. (as well as *Chlorella* and *Scenedesmus* spp., again) may be able to survive and even flourish in the (pretreated) produced water [53].

## 3. Antimicrobial Metabolites from Microalgae and Cyanobacteria

It is well known that free fatty acids (FFAs) have a general antibacterial effect [54]. There are reports that the maximal content of FFAs in algae culture medium is in the stationary phase and is directly correlated to the process of autolysis. That could be due to their release after cell lysis and could also happen in natural conditions in the aquatic environment. These compounds could secure the resistance of the natural biotope to settlement of allochtonic microbes and stability of the structure of the biocenose. Antibacterial compounds are also synthesized as a result of chlorophyll and FFA oxidation. A protein–chlorophyll complex of *D. maritima* inhibits the growth of the bacteria *Pseudomonas saccharophila.* This complex could also be released after cell lysis, and that could also happen in the water basin. The velocity of elimination of foreign microorganisms could rise at microalgal mass dying, and this is one of the mechanisms of self-purification of water bodies. The antibacterial effect of peloid (mud or clay often associated with water basins and used for therapeutic purposes) could be largely due to the intracellular compounds released during microalgal destruction [55,56].

Fatty acids, especially PUFAs, are one of the most abundant and highly antibacterial compounds in microalgae and have an underappreciated antimicrobial therapeutic potential [57]. The double bonds of unsaturated fatty acids are claimed to contribute to higher antimicrobial activity in comparison with the saturated fatty acids [58]. Their mechanism of action is mostly related to their surfactant nature, leading to cell membrane damage and cell and electron leakage. They also inhibit electron transport systems, ATP production and other bacterial enzymes and induce peroxidative reactions [59,60,61,62,63,64,65,66]. EPA and DHA have infection-suppressing properties in vivo [67]; however, as noted, *Arthrospira* and *Dunaliella* are not rich sources of them. Nevertheless, *A. platensis* is rich in the antibacterial γ-linolenic acid (36% of the total PUFAs) [32]. However, this acid has a high activity only against Gram-positive bacteria but not against Gram-negative ones [58].

Other lipids, as well as polysaccharides and other carbohydrates, can also have antimicrobial properties [3,60,68,69]. *Dunaliella* spp., as well as other eukaryotic microalgae, such as *Chlorella* spp., produce and secrete relatively large amounts of antibacterial polysaccharides (PSs) [3,60]. The pigments chlorophylls and carotenoids, especially β-carotene, are also effective microbial growth inhibitors [70,71]. PSs, e.g., sulphated exoPSs, are also surfactants and have anti-adhesive properties against microbes. That may be due to their competing with carbohydrates (glycans) on host cell surfaces, which are the recognition sites for bacteria to attach to using their lectins on the bacterial surfaces [72,73]. PSs binding with bacterial glycoprotein receptors leads to increased cell permeability and bacterial DNA binding [59,74]. The biofilm-preventing effect is related to prevention of bacterial autoaggregation, which is also lectin-dependent [75,76].

Other major antimicrobial (antibacterial, antifungal and antiviral) compounds of *Arthrospira* are phycocyanin, phycocyanobilin and allophycocyanin, and they have certain anticancer effects as well [69,77]. The taxon possesses also flavonoids and other phenolic compounds and terpenes, which are proven antimicrobials [69,78]. Phenolic compounds at high concentrations can denature proteins in bacterial cell walls and membranes through hydrogen bond formation, thus damaging them and causing lysis due to leakage [60,79,80]. At low concentrations, they likely influence enzymes, especially those involved in energy production [68]. The antifungal effect may be due to binding and damaging lipids in cell membranes and organelles and the impact on spore germination [81,82]. Terpenoids are capable of damaging the porins in the outer membrane of bacterial cell walls through the formation of a strong bond polymer, thus impeding the flux of nutrients [66]. *A. plantentis* also has alkaloids, saponins and quinones. Alkaloids can interfere with components of peptidoglycan in bacterial cells wall, thus damaging the wall and causing cell death. Saponins are detergents which can decrease the voltage between the bacterial cell wall and permeabilize the membrane. They reduce the surface tension of the cell wall, thus penetrating the cell, disrupting cell metabolism, and ultimately causing the bacteria to die. Quinone compounds can bind to cell proteins, rendering them dysfunctional, thus disrupting cell metabolism [66,79].

Microalgal steroids destroy the bacterial cell membrane [66]. Microalgae, including *Dunaliella*, owe their antimicrobial properties also to carotenoid degradation products such as the monoterpene β-cyclocitral and the sesquiterpenes α and β-ionone, neophytadiene, the diterpene phytol [60,79,80,83], polyunsaturated aldehydes, bromophenol, glycosides and peptide structure classes [84,85]. The hydrocarbon neophytadiene from the phytane family has been detected in high amounts in antimicrobial essential oils from tobacco leaves [83].

**Figure 2:**
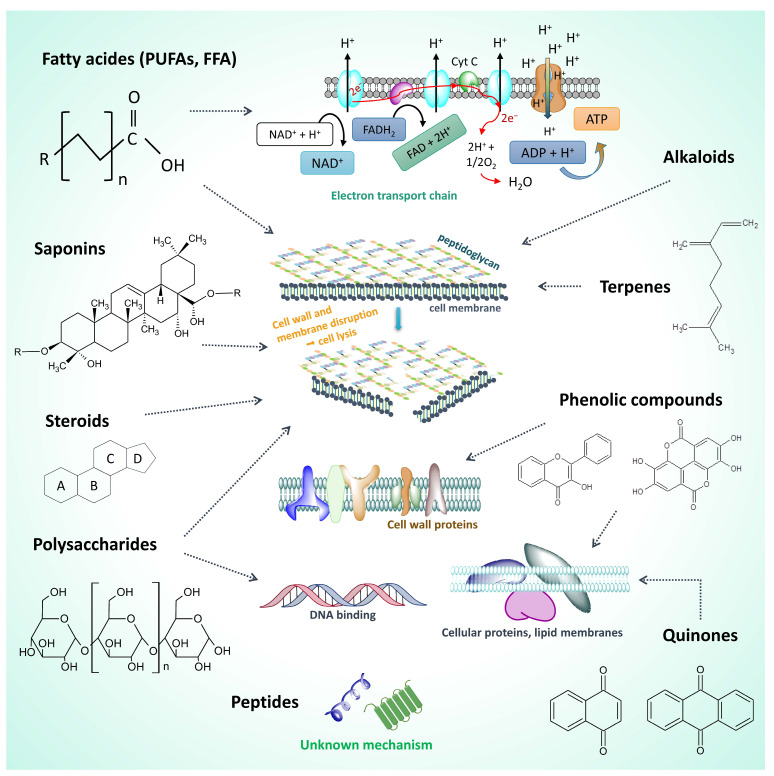
Examples of mechanisms of action of antimicrobial microalgal and cyanobacterial metabolites. Legend: PUFAs—polyunsaturated fatty acids, FFA—free fatty acid.

Phytoplankton is an emerging source of antibacterial peptides. For example, all cyanobacteria, including *Arthrospira* are reported to be able to synthesize the antimicrobial toxins microcystins, which are cyclic heptapeptides. They are produced in large quantities during algal blooms and are a major threat to drinking and irrigation in terms of toxicity. While this does raise safety issues associated with commercial spirulina products, the potential of microcystin as an antibacterial agent can be explored [79], similarly to how they are explored as cancer drugs [86]. Antibacterial peptides are mainly cyanopeptides, meaning they are mostly in cyanobacteria, such as Oscillatoriales and Nostocales [69,87]. Apart from microcystin, few antibacterial peptides have been isolated from *A. platensis* [88,89]. The mechanism of action of cyanobacterial and microalgal peptides is not generally known [69]. It is interesting that *Arthrospira* spp. are one of the marine microbial species that contain intracellular polyhydroxybutyrate (PHB) polymer. There are many reports demonstrating that the PHB particles are stored as carbon and energy reserves under severe stress conditions. PHB has exerted a strong inhibitory and anti-adhesive effect against *Vibrio* species pathogenic to shrimp and *Nile tilapia* fish [90]. Not only can it be used as an effective antimicrobial agent, but it could present opportunities to be used as a packaging polymer [91]. *Arthrospira* spp. also has antimicrobial glycosides [78] and gallic and caffeic acids, which generally have an antibacterial effect [91]. Other specific antibacterial compounds from phytoplankton are listed in the work of Rojas et al., 2020 [69]. An illustration of most of the known mechanisms of antimicrobial action of microalgal and cyanobacterial metabolites is presented in Figure 2.

## 4. Maximizing the Synthesis of Bioactive Metabolites including Antimicrobial Compounds by Phytoplankton Microorganisms, Involving Stress Factors

### 4.1. The Two-Step Process

Theory in the field is well established, and the two-step process of maximization of secondary metabolites is well studied and proven.

The first step considers the working condition under which the microalgal growth is kept close to the maximum growth rate by applying well-balanced and rich medium where no limitation of growth by nutrients may occur. Hence, microalgal cells’ growth in non-limiting conditions in the logarithmic phase up to the stationary phase is supplied by keeping state parameters (CO_2_ content, pH, T, pressure, light intensity, mixing conditions, changing organic source of carbon, etc.) in optimal values. When the culture reaches the stationary phase, usually secondary metabolites are synthesized under stress factors, which directs metabolism of microalgae to their overproduction. Most common manipulation of microalgal metabolism is under stress conditions, such as nutrient starvation, high salinity, high temperature, light stress and supplementation of organic carbon source or by using several stress factors together. The results can be production of high-value compounds (for example, lectins) expressing anticancer, antiviral, antibacterial and anti-oxidant activities. Especially important to notice is that in open ponds, biomass concentration is low and production of secondary metabolites is low as well. Hence, application of closed photobioreactors (PBRs) is advanced and provides many benefits, such as high-density culture, controlled process conditions and precise manipulation of stress factors directing the metabolism of microalgae to over production of bioactive compounds. Studying PBRs performance as a complex system is fundamental for overall process development [92,93].

### 4.2. Stress Factors Such as Salinity, Nutrient Deficiency, Temperature and Light Increase Carotenoids and Other Metabolites in Dunaliella

Of special interest is the synthesis of carotenoids [94,95]. The main microalgal carotenoids are astaxanthin, β-carotene and lutein. Many microalgal species are known to accumulate these carotenoids; however, the main species that are extensively studied are *Dunaliella salina, Haematococcus pluvialis*, *C. zofingiensis* and *Chlorella vulgaris* due to their capability of commercial production in large scale cultures. Secondary carotenoids synthesis is affected by the variation of the cultivation settings, and their accumulation is induced by the exposure of the cells to several stress factors [96]. Carotenogenesis is enhanced by reactive oxygen species (ROS), which are generated by stress conditions like high light intensity, salt stress or high temperature [97].

*D. salina* contains β-carotene typically about 0.5–1% of dry weight, as many other green microalgae [98]. However, as noted, under stress cultivation conditions, β-carotene is accumulated within lipid globules in the chloroplast to more than 12% of the algal dry weight, which is the highest content of β-carotene of any known source, making *D. salina* the best source of β-carotene [98]. The β-carotene accumulation and the rate of synthesis depend on certain environmental parameters [99]. As in the case of astaxanthin, β-carotene accumulation in *Dunaliella* sp. is also primary triggered when the cell division rate is either slowed down or arrested due to the effect of the stressing factors [100]. Nutrient starvation in cultures of *D. salina* is well investigated, and it is well known that under nitrogen, phosphorus and sulfur starvation, the cells have elevated content of β-carotene [101]. Between sulfur, nitrogen and phosphorus starvation, the highest increase of β-carotene accumulation was obtained with phosphorus starvation [102]. However, in the large and commercial-scale production of β-carotene by *D. salina,* the technique used is high-salinity stress and nitrogen deficiency [103]. In nitrogen starved cultures of *D. salina,* the β-carotene content increased up to 2.7% of ash free dry weight from about a content of 0.75% in nitrogen replete cultures [100]. It was observed that in nitrogen-starved cells, total fatty acid content did not increase, but only an increase of specific fatty acids occurred [100]. In contrast to lipids, *D. salina* under nitrogen starvation accumulated carbohydrates over 55% of dry weight, while its total protein content along with lipids decreased [104].

*D. salina* is able to grow under a very wide salinity values from 0.5 to as high as 35% *v*/*w* NaCl [105]. The majority of the *Dunaliella* species have optimal growth in cultural medium containing 1–2 M NaCl. Salinity tolerance is positively linked with temperature of the liquid phase. For example, at 15 °C, the cells grow in about 1 M, and at over 30 °C, the culture adapts to over 3 M [106]. Certainly, increasing the salinity in the culture has to follow a particular strategy in order to achieve optimal results [107]. On the other hand, salinity stress is responsible for over-production of carbohydrates in *D. salina*. If the medium contained 5.5 M salt, the culture reportedly synthesized 2.5-fold more carbohydrates [108].

Decreasing the culture temperature from 30 °C to 10 °C resulted in a 2-fold increase of β-carotene value [109]. It has to be noticed, that cultivation in low temperature increased the 9-cis-β-carotene and α-carotene value of the cells [110]. Nutrient deficiency, for example, nitrogen starvation, resulted in an increase of carbohydrate content up to 40% [104].

Microalgal culturing under light illumination stress conditions is a challenging method for β-carotene over-synthesis. The effect of light changes has a positive influence of production of β-carotene [111]. In cultures of *D. salina*, transferring the light conditions from 100 to 1000 μmol photons m^−2^ s^−1^ resulted in a significant increase in β-carotene content, where the maximum at 3.1% of dry weight was achieved [112]. However, combined light stress and nitrogen starvation conditions seem to result in more accumulation of β-carotene [100,112]. Cellular β-carotene accumulation due to light stress results in accumulation of specific fatty acid species (C16:0 and C18:1) and especially of C18:1, while total fatty acid content is not significantly affected [112]. The degree of light intensity affects also the isomers of β-carotene; low light intensity favors the synthesis of the 9-cis isomer, while high light favors the all trans β-carotene. It has to be noted that light illumination in a particular wavelength influences the β-carotene synthesis, for instance, blue light [113]. It is interesting that, recently, application of closed PBRs led to very promising results with some other microalgal species (eustigmatophytes) regarding β-carotene accumulation [114,115]. Many frontiers for optimization of secondary metabolites over-production are achieved by using mixoptrophic and heterotrophic growth of microalgae. Strategies for such approaches can be found in the special literature about the mode of microalgae cultivation.

### 4.3. Optimization of Metabolites from Arthrospira spp.

The influence of light wavelength on *A. platensis* metabolism is studied in detail by Sohani et al., 2023 [116]. They investigated cell behavior under blue, red, yellow and white light illumination.

Especially important once again to be noted is that manipulation of environmental factors as stress ones is based on a robust theory about methodology and established principles of optimization of cell cultivation. An excellent example can be given by citing the work of Chentir et al., 2018 [117]. In their work, the established methods of successful culturing are applied in order to achieve intracellular and extracellular valuable compounds. Using statistical methods and the effect of applying stress factors such as light intensity, NaCl, nitrogen and phosphorus, they succeed in maximizing intracellular and extracellular substance produced by *Arthrospira* sp. Certainly, the working conditions were realized in a two-phase cultivation process. The carbohydrate content was maximized up to 26.6%. Using this approach, the maximum content of lipids (15.6%) was achieved.

The conclusion from this work is fundamental and demonstrates that manipulation and combination of the multiple stress factors together with application of advanced culturing techniques such as the multi-step culturing process is the only winning strategy which saves time and resources for research. The strategy can be applied for any microalgal species by taking into account specific requirements of its metabolism and is thought to be a promising tool to produce biomass enriched in various high-value compounds.

## 5. Immunomodulatory Activity

Microalgae, cyanobacteria and their compounds have great ability to fight and prevent infections indirectly through their immunostimulating properties. Carotenoids and vitamins have immunological activity, e.g., the relationship between vitamin E supplementation and membrane lipid protection from oxidative damage has been the subject of numerous studies. This is largely due to the fact that carotenoids and many vitamins are antioxidants having general immunostimulant activity [118]. As noted, the proteins, B12 and other vitamins from *Arthrospira* are used for antioxidants and immune enhancers [36]. *Arthrospira platensis* is rich in β-carotene, essential fatty acids, vitamin E, B12 and other vitamins and minerals, which makes it a great antioxidant and immune enhancer. In fact, not all immunological compounds from *Arthrospira* are elucidated, but the genus, as well as some microalgae, has proven immunological activity due to the activation of the innate immune system by increased interferon production and natural killer (NK) cytotoxicity in humans [118,119]. For instance, oral administration of hot water extract of spirulina significantly increased the production of interferon-γ in NK cells [120].

As noted, PUFAs have anti-inflammatory action. They, together with phytosterols, carotenoids and vitamins are the most explored phytoplankton compounds for their effects in the regulation of immunological responses. *Dunaliella tertiolecta* and *D. salina* are rich in phytosterols, giving the species an immunomodulatory potential [118]. Indeed, *D. tertiolecta* and *D. bardawil* have been reported to have anti-inflammatory properties. *D. salina* activates natural killers and macrophages in mice and interleukin (IL)-6 in human peripheral blood mononuclear cells (PBMCs) [119]. A mixture of phytosterols from *D. tertiolecta* inhibits the proliferation of PBMCs and cytokine production in a sheep model of inflammation [118,119].

As we noted, some anti-inflammatory metabolites, such as carotenoids, fatty acids and sulphated polysaccharides, have also been reported to increase after various stress chemical triggers in *A. platensis*, *D. salina*, *C. zofingiensis,* etc. [37].

The two genera assessed in the current review have a clear non-specific immune response enhancing effect in shrimp and fish. Thus, they have vibriosis preventing and *Aeromonas* infection preventing effect. While it is certain that it is largely due to their immune stimulating effect and immune prophylactic [121] action, it is also very likely that their antibacterial activity has a role [85]. *Artemia* (brine shrimp) fed with a daily supplementation with two strains of *D. tertiolecta* were fully protected against vibriosis after challenge with *Vibrio* sp. in terms of survival and viable parameters. The results were better than when using probiotics and dead bacteria. It is not known whether this effect is due to the immune stimulating effect or the synthesis of antibacterial compounds [122]. Carp that were fed with *A. platensis* had an enhanced response of phagocytosis, superoxide anion production and IL-1β and tumor necrosis factor (TNF)-α genes expression in kidney phagocytic cells [123]. Phagocytes from channel catfish fed also with *A. plantesis* showed enhanced phagocytic activity to zymosan and increased chemotaxis to *Edwardsiella ictaluri* exoantigen [124].

Dry powder and hot water extract of *A. platensis* increased resistance against *Vibrio alginolyticus* in the white shrimp *Litopenaeus vannamei* due to increased phagocytic and lysozyme activities for pathogen clearance [121,125,126]. Superoxide dismutase was induced and activated, and this is an essential antioxidant enzyme playing a vital role in the immune system of shrimp in scavenging superoxide anion that damages the host tissues [121,125]. All shrimp that survived vibriosis in the study of Al-Ghanayem (2023) developed resistance to *Vibrio* pathogens, which is explained by the fact that antioxidant enzyme levels of shrimps had increased after treatment with *A. platensis* extract and antioxidant enzymes increase immunity patterns of the host. According to this study, *Arthrospira* bioactive compounds have the potential to function as both inexpensive and environmentally friendly immune stimulants as well as an effective preventive measure against *Vibrio* bacterial infections [121].

## 6. Antimicrobial Activity of *Arthrospira* spp.

### 6.1. In Vivo Antimicrobial Activity

It is noteworthy that a substantial amount of in vivo research (Table 1) has been dedicated to *Arthrospira* in comparison to other phytoplankton, such as *Chlorella*, or generally the former *Chlorella* genus, *Scenedesmus*, *Dunaliella*, etc.

An example of such research is about impetigo. This is a highly infectious skin bacterial disease, most common among pre-school children, and is mostly caused by *Staphylococcus aureus*. Gas chromatography-mass spectrometry (GC-MS) analysis of both crude cell biomass and its methanol (MeOH) extract from *A. platensis* detected a high percentage of linoleic and palmitic acid and the presence of squalane in the MeOH extract, which could act in synergy, resulting in impetigo infection suppression [8].

*Arthrospira platensis* had an in vivo antimicrobial effect on the bacterial groups investigated in the rabbit cecum but only in combination with thyme. The qPCR detected significantly lower bacterial load in the samples, while the classical microbiological methods did not measure a substantial effect on the composition of the cecal microbiota. Samples were collected on the 14th, 28th and 48th day of the supplementation (a 48-day growing period). The number of *Escherichia coli*, total anaerobic and strictly anaerobic bacteria decreased by age, regardless of the diet type. There was a drawback: *A. platensis* plus thyme had a negative effect on cellulose and on crude protein digestibility, impairing the rabbit diet’s digestible protein content [127]. The authors suggested testing the effects of *A. platensis* and/or thyme on health status under poorer sanitary conditions [128].

Vibriosis is a common lethal bacterial infection in shrimp in hatcheries and farms. *Vibrio* species amount for more than 80% of the bacterial population in seawater and are opportunistic pathogens infecting shrimps under environmental stress [121,129]. *V. alginolyticus*-infected shrimp are more susceptible to other pathogens and ultimately dying. *Vibrio harveyi* is a luminescent bacterium that causes luminous vibriosis and is among the most common pathogens that increase the mortality in aquaculture shrimp species, such as the white shrimp *L. vannamei* [130]. *Vibrio parahaemolyticus* is also a major pathogenic strain for *L. vannamei* and causes acute hepato-pancreatic necrosis disease, a.k.a. early mortality syndrome, which is a relatively new disease that emerged in Southeast Asia in ca. 2010 and may cause 100% mortality within 20 days of infection [131,132]. The AMR in *Vibrio* spp. towards antibiotics in aquaculture and antibiotic residues left in water are also pressing problems [133].

The in vivo study of Al-Ghanayem (2023) utilized tests, one of which included separate suspensions of the three *Vibrio* species mixed with a MeOH *A. platensis* extract intramuscularly injected in an abdominal segment of 12 white shrimp. The positive control inoculated shrimps were severely infected within 72 h and died within 120 h, except the *V. harveyi*-infected shrimp, which survived slightly longer. *Vibrio alginolyticus* was the most virulent. A challenge test included shrimps fed with 2500 μg/g extract of *A. platensis* and injected with suspensions of the three *Vibrio* species separately into the ventral sinus of the cephalothorax. The survival rate was recorded every 24 h for 168 h. The two assays clearly showed increased survival rate and reduced shrimp mortality and demonstrated that *A. platensis* considerably influenced pathogen multiplication regulation. Apart from the immunostimulant action, which contributes to overcoming environmental stress, the direct antibacterial activity could also contribute to the results. The conclusion is that *A. platensis* could effectively prevent *Vibrio* infection and considerably protected *L. vannamei* against vibriosis [121].

Doses of 1 mg, 10 mg or 25 mg *A. platensis* per fish were administered orally to carp by intubation for 3 days. After *Aeromonas hydrophila* challenge, fish were sacrificed and the livers and kidneys were removed, pooled, homogenized and diluted. Suspensions were plated on ager and the number of colonies was counted after 12 h. The results showed that the numbers of bacteria in the liver and kidney in the control group peaked at 8 and 1 h, after the challenge, respectively, and then decreased on their own. However, *A. platensis* feed aided that process. The kidneys of the treated groups had lower bacterial numbers than the control only at the early hours (1–4), while the liver of the treated groups had lower bacterial numbers at hours 4–12 [123].

Wound assays have a considerable part in the in vivo research regarding *Arthrospira*. An example of these reports used 30 rats and a clinical in vivo wound procedure with contamination with *Pseudomonas aeruginosa* and *S. aureus* for 24 h before treatment. The signs of inflammation were more obvious with *P. aeruginosa*. The negative control (without treatment) showed partial healing and shrinkage in the wound area but also formation of scar tissue after seven days. Treatment with the aqueous extract or with standard antibiotics (azithromycin for *S. aureus* and ceftazidime for *P. aeruginosa*) showed comparable results. In comparison with the untreated group, there was a complete or accelerated healing, sometimes without remnant scar tissue. Wound shrinkage and a process of re-epithelialization were observed. All this is a reflection of a good antibacterial activity, as inhibition of bacteria in the wound site is, authors say, probably what led to cell proliferation and regeneration. However, the antibacterial activity is not the only cause of healing, which might be attributed also to stimulation of angiogenesis, collagen formation and deposition, and epithelial cell proliferation. Flavonoids, alkaloid, terpenes, glycosides and saponins but not tannins were found in the extract, and some of them are not only antimicrobials but antioxidants. Since antioxidants are also reported to have a significant role in the wound healing process by protecting tissues from oxidative damage, antibacterial and antioxidant compounds might act synergistically in the wound healing process [78].

*Arthrospira platensis* cream of a MeOH extract proved to be better that a nystatin cream for murine wounds infected with *Candida albicans*. The wound healing recorded for nystatin was 50–90% from days 4 to 13 after treatment. However, *A. platensis* cream caused wound healing of 55–95% for the same time interval. At the 13th day, the wound’s redness was at its lowest and hair growth was moderate; by the end of the 17-day study period, all inflammation had disappeared and there was significant hair growth. The histopathological analysis of the untreated wounds showed several missing layers of the epidermis, rounded, short, elongated cells, hypha swelling and keratinized fiber that were loose and disordered in the stratum corneum. The epidermis also had a chronic inflammatory cellular infiltration mainly of lymphocytes and plasma cells. The nystatin-treated wounds still had abnormal epidermis as the keratinized fibers of stratum corneum were also disrupted and had separation of the epidermis. The dermis also had edema with some inflammatory lymphocyte infiltrate. Mice treated with *A. platensis* showed a finding similar to the healthy skin, with no hypha swelling, no discernible toxic consequences compared to the control, normal looking skin tissue and epidermis, normal dermis with minimal inflammatory cellular infiltration and with keratinized fibers of the stratum corneum consistently and regularly arranged and condensed without any disruption. The high antioxidant activity of the MeOH extract could also contribute to the wound healing effect. Alkaloids, phytol, fatty acids hydrocarbons, phenolics and phthalates were found in the extract [134].

Chlorophyll is a natural photosensitizer, and when irradiated with a laser, it generates reactive oxygen species (ROS), thus causing photodynamic destruction of bacteria, for example, in an infected area. This interesting and innovative phenomenon was developed by Li et al., 2021, [135] for *A. platensis* coated with a formulated natural chitosan polymer. The hydrogel promotes the adhesion to wounds and the cyanobacteria release chlorophyll, which is irradiated with a 650 nm laser. However, the main function of the photosynthetic cyanobacteria was the production and local delivery of oxygen to alleviate acute and chronic tissue hypoxia in wounds. This combined action enhances wound healing. With increasing time of laser irradiation, the ROS level increased, demonstrating the laser-induced ROS participated in the antibacterial process. *Arthrospia platensis* with a 650 nm laser could significantly inhibit *E. coli* and *S. aureus* growth in vitro, with over 80% antibacterial efficiency, compared to the control. In vivo, comparing with the untreated control, the groups of chitosan, SP gel and SP gel + laser showed enhanced wound healing, which was slowest for the chitosan-treated mice. Laser irradiation could significantly enhance the effect and prolong the activation time of *A. platensis* gel in the wound healing process and could satisfy the oxygen requirement for wound healing, leading to best neovascularization. The authors named the formulation a living hydrogel, which relies on both photosynthetic ability and photodynamic effect to inhibit infection, alleviate hypoxia and accelerate wound closure and is promising for clinical translation [135].

The same mechanism was developed in another study; however, *A. platensis* in the chitosan gel was loaded with the plant alkaloid berberine, a quorum sensing inhibitor and antibacterial agent. All in vitro and in vivo antibacterial results, i.e., in wound healing, were the synergetic effect of laser-irradiated *A. platensis* and berberine, therefore not a subject of this study [136].

In an animal study feeding mice with phytoplankton agents and *Helicobacter pylori* inoculation, the short-term treatment was repeated for two consecutive days. To study the effect of a longer period, other mice were fed either with polysaccharides or 35 mg of *A. platensis* powder 3 times per week for 4 weeks before infection with *H. pylori*. All mice were sacrificed two weeks after the last treatment, and the stomach of each animal was removed for further analysis. *Helicobacter pylori* that were present in it were isolated, identified and enumerated. Treatment of mice with the polysaccharides only up to 2 h before inoculation with *H. pylori* significantly reduced the mean bacterial load in their stomach by more than 50%. *Arthrospira platensis* powder had a positive but not statistically significant effect. When the mice were fed with polysaccharides and powder for 4 weeks before infection, reduction in *H. pylori* load was significantly and more obviously reduced by 94% and 87%, respectively. Doses of twice the effective inhibitory concentrations used in an in vitro inhibition study did not kill *H. pylori* and AGS gastric epithelial cells. A polysaccharide agglutination assay showed that allowing porcine gastric mucin to interact with the polysaccharides before the addition of *H. pylori* did not prevent the agglutination of *H. pylori* by them. Similarly, the presence of mucin did not impede the polysaccharides in agglutinating *H. pylori*. Polysaccharides from *A. platensis* were confirmed to be effective carbohydrate-based antiadhesives in preventing *H. pylori* adhering to gastric mucin and colonization without affecting the host [137].

Cold atmospheric pressure plasma (CAPP) technology is currently considered an effective and cost-effective new technology to sterilize food and pharmaceutical matrices in a few minutes. However, more research is required in relation to food–plasma interaction and food macromolecules stability/functionality under the new CAPP technology. Pina-Pérez et al., 2022, [138] conducted an in vivo assessment of this technology on the bioactive activity of *Arthrospira* powder using *Caenorhabditis elegans* as an animal model. This nematode has been used very frequently as a model of infection by pathogenic microorganisms, whereas survival of infected worms, either exposed to a tested antimicrobial agent or not, is assessed. CAPP did not have negative effects on the nematode, and both CAPP-treated and untreated *A. platensis* improved *C. elegans* lifespan. On the topic of the current review, no antimicrobial effect, measured as an increase in *C. elegans* lifespan, was detected in worms infected with *Salmonella enterica* serovar Typhimurium when CAPP-treated or untreated *Arthrospira* was added to the media. Approximately half the worms of all groups were dead at ca. 12 days [138].

**Table 1:** Research which included studying the in vivo antimicrobial activity of *Arthrospira platensis* Gomont.

Extract or Sample	Test Microorganism or Animal Model	Antibacterial Activity		Test Methods	Reference
		In Vitro: Minimum Inhibitory Concentration (MIC)/Minimum Bactericidal Concentration (MBC)/Inhibition Zone (IZ) and Mode of Action	In Vivo		
Crude extract (biomass) MeOH extract Creams from both	Sensitive and methicillin-resistant *Staphylococcus. aureus* Patients with impetigo	MeOH extract > crude extract	MeOH extract—best efficacy, no side effects, no recurrence during the follow-up period	Agar well diffusion method (AWDM) Topical application of creams	[8]
Feed pellet with 5% *Arthrospira platensis* (S), Feed pellets with 3% thyme (T) or both (ST)	Pannon white rabbits	-	ST > S and T Decreased number of *Clostridium coccoides* and *C. leptum* in the caecum	Dietary supplementation for 48 days PCR for bacterial enumeration	[127]
Aqueous extract 100 mg/mL	*S. aureus* *Pseudomonas aeruginosa* *W*ounds in rats (either pathogen)	19 mm IZ 18 mm IZ	Accelerated epithelial regeneration comparable to control antibiotics	AWDM In vivo open-wound model (3 cm incision, 7 days treatment)	[78]
EtOH extract MeOH extract Acetone extract EtOAc extract	*Candida albicans* *Malassezia furfur* *Trichophyton rubrum*	17–19 mm IZ for the MeOH; 16 or lower for the acetone and EtOAc extracts; 10–12 mm for the EtOH extract Mode of action: cell lysis	Elimination of *Candida* spherical plastopores from skin layer after application of cream with MeOH extract	AWDM: 100 µL of 5% extracts MIC assay on agar TEM In vivo *C. albicans* infection in mice	[134]
Hot water extract	White shrimp *Litopenaeus vannamei* challenged with *Vibrio alginolyticus*	-	6–20 μg/g (injection) or seawater with 400–600 mg/L (24–96 h culturing): enhanced phagocytic activity, better clearance efficiency and survival rates	Challenge test by injecting bacterial suspension into the ventral sinus of the cephalothorax of *L. vannamei*	[125]
MeOH extract	*V. alginolyticus* *Vibrio harveyi* *Vibrio parahaemolyticus* *L. vannamei* shrimp infected with *Vibrio* culture	MIC/MBC [mg/mL]—2/2.5 1.5/2 1.5/2	Increased survival rate: 33, 50 and 17% for *V. parahaemolyticus*, *V. harveyi*, or *V. alginolyticcus*-challenged shrimps, compared to 0% for the group with no extract (feed test); survival of 16–25% (mixed injection test); full eradication of *Vibrio* spp.—RT-PCR (−)surviving shrimps	BMD assay (MIC); Agar plate test (MBC) In vivo model of vibriosis in *L. vannamei*: mixed injection of *Vibrio* sp. and extract and challenge test (extract in the feed)—168 h monitoring RT-PCR	[121]
Dried powder	*L. vannamei* challenged with *V. alginolyticus*	-	Dose-dependent effect against *V. alginolyticus* Survival rate after 144 h: 65% (60 g/kg powder), 50% (30 g/kg) and 38% (positive control)	Injection of *L. vannamei* with bacteria (ventral sinus of the cephalon-thorax: four-week diets (0, 30, 60 g/kg powder), survival rates every 24 h	[126]
Physiological saline suspension of cells	The carp *Cyprinus carpio* infected with *Aeromonas hydrophila*.	-	Decreased numbers of *A. hydrophila* in liver and kidney of *A. platensis*-fed fish.	Intraperitoneal injection with 3 × 10^7^ colony-forming units (CFUs) of *A. hydrophila* per fish	[123]
Free *A. platensis* (AP) Free carboxymethyl chitosan hydrogel with *A. platensis* Combination with 650 nm laser irradiation	*S. aureus* *Esherichia coli* Murine wounds infected with *S. aureus* and suppurated	AP: 350 μg/mL reduces CFU to 63% (*E. coli*) and 41% (*S. aureus*); AP + Laser (1 w/cm^2^, 10 min): 10 to 19% CFU; Induction of ROS	AP gel + laser: highest efficacy, less inflammatory cells, milder inflammation	CFU plate counting method in vitro In vivo wound model in mice: 10 mm wounds immunohistochemistry	[135]
Polysaccharide extract, powder	*Helicobacter pylori* *H. pylori*-inoculated mice	Mode of action: binding of *H. pylori* alkyl hydroperoxide reductase and urease; agglutination of *H. pylori*	35 mg prevents by 99% binding of *H. pylori* to gastric mucin at pH 2.0 if applied 1 and 2 h before infection Reduction of *H. pylori* load by >90% in mice	Competitive and blocking inhibition assay Ligand overlay analysis Agglutination assay In vivo mice model (10^6^ CFU of *H. pylori*)	[137]
*A. platensis* treated or untreated with cold atmospheric pressure plasma (CAPP)	*Salmonella typhimurium*-infected *Caenorhabditis elegans*	-	*A. platensis* (1 mg/mL CAPP untreated or treated) does not present in vivo effect against *S. typhimurium*	In vivo model: cultivation of *C. elegans* with bacteria, synchronization of worms at larval stage L4	[138]

### 6.2. In Vitro Antimicrobial Activity

In vitro reports on the antimicrobial properties of *Arthrospira* are the most numerous in the current review (Table 2 and Table 3).

Non-polar extracts from the species were found to have higher antimicrobial activity than polar ones in one report [68]. Atomic force microscopy (AFM) showed that one hour of exposure to an acetone-soluble fraction from *A. platensis* cells caused considerable damage to cell walls of *A. hydrophila* bacteria—pores, holes and grooves had formed. After two hours of exposure, the cells had become permeabilized and collapsed due to fragmentation of the cell wall [139].

Many aquatic pathogenic bacteria use quorum sensing system to establish their virulence [140], and *Vibrio* spp. are one of them. Co-culturing of *A. platensis* and different *Vibrio* spp. leads to positive results—high inhibition of bacterial growth. This antibacterial activity was not influenced by the presence or absence of light as well as for other phytoplankton species. One potential course of action might involve interfering with quorum sensing. Previous studies have demonstrated the high sensitivity of *Vibrio* to these mechanisms. The findings from that study could provide an explanation for the beneficial effects of adding phytoplankton to fish larvae generally [85].

De Souza et al., 2011, [141] found out that the dry mass of *Aspergillus flavus* was on average 4× lower in the presence of phenolic compounds from *A. platensis* than in the control. They analyzed the indicators of *A. flavus* biomass development (glucosamine, ergosterol and protein content) and concluded that glucosamine content is the most suitable to investigate the effect of phenols on the fungal biomass development because its production was linear along the time in the control experiment and was the most affected in the culture medium. The membrane component ergosterol was hardly affected, and fungal biomass protein content had an unstable pattern of production over time [141].

Biofilms are held together and covered by a matrix made up of strong biopolymers called extracellular polymeric substance (EPS). A decrease in EPS will cause the biofilm structure to weaken, making the bacteria living there more susceptible to antimicrobial agents and simpler to remove. Bacterial cell surface hydrophobicity (CSH) plays a crucial role in the initial bonding of the bacterial cell for the first few seconds in the biofilm. Weakening of CSH would reduce the initial attachment and leads to decreased bacterial population in the biofilm. Therefore, according to LewisOscar et al., 2017, [7] an ideal antibiofilm strategy has to control these factors (CSH and EPS). *Arthrospira platensis* extract achieved biofilm inhibition and significant reduction in CSH in some bacterial species. In addition, the biofilm inhibitory concentration did not exert antibacterial activity and was not bactericidal. This makes the cyanobacterium a potential candidate for antipathogenic and antibiofilm agents, less prone to induce antimicrobial resistance [7].

As noted, the PHB from *Arthrospira* spp. could be used as an antimicrobial therapeutic and for the production of packaging polymers [90,91]. The PHB from *Spirulina* sp. LEB 18 is biodegradable and has similar mechanical and thermal properties to polymers of petrochemical origin. PHB nanofibers were prepared by injecting polymer solution of PHB through capillaries with a diameter of 0.45–0.8 mm. That solution contained phenolics extracted from the biomass of the same species, namely gallic (72%) and caffeic acids (28%), which generally have an antibacterial effect. This material (35% PHB/1% phenolic compounds), albeit not highly active against *E. coli* and *S. aureus*, showed retaining of the phenolics’ activity after the process of electrospinning. This new nanomaterial combined a biodegradable packaging function with the antibacterial activity that prevents the multiplying of microorganisms and ensures the quality and preservation of food [91].

The active ingredients from *A. platensis* with proved antimicrobial activity were found to contain some antimicrobial compounds. Naturally, high content of long-chain fatty acids [142], e.g., in non-polar extracts [68], other fatty acid compounds [143], γ-linolenic acid and the synergetic effect of lauric and palmitoleic acid [144,145] have been considered responsible for the antibacterial action. Other compounds included proteins, carbohydrates, flavonoids, phenols, steroids [146,147], alkaloids, saponins, phenols, quinones (from an ethanol (EtOH) extract) [79], terpenes (monoterpenes and sesquiterpenes) [146,148] and glycosides [147]. A positive correlation between the antimicrobial effect and the phenolic content in the respective extracts has been found in a study, and the more active extracts, the non-polar ones, contained lipid-soluble phenolics [68]. In another study, the phenolic compounds did not turn out to be the main component of the most active samples [88].

The peptides with <3 KDa molecular weight from the species inhibited *E. coli* and *S. aureus*. As previous studies had shown that only peptides with <6 KDa molecular weight can pass through gastro-intestinal epithelial cells [149], the isolated peptides should be capable of being developed as oral drug candidates. In another study, the antibacterial substance was indicated to be an aliphatic compound with different active groups (–OH, =C=O, =CH_2_ and –CH_3_) [150].

**Table 2:** Research on the in vitro antimicrobial activity of *Arthrospira platensis*.

Extract or Sample	Test Microorganism	Antibacterial Activity: MIC, MBC, IZ, etc.	Test Methods	Reference
Ethanol (EtOH):water (70:30) extract	*E. coli, S. aureus, Bacillus cereus, Listeria monocytogenes* *Salmonella* spp. (isolates from pigs), *Salmonella enterica* (incl. serotype Rissen)	Most effective after 24 h treatment IZs from 9 to 15 mm (most active against *B. cereus, L. monocytogenes*, *S. aureus)* Bacteriostatic activity at 0.45% (*v*/*w*) Bactericidal effect at 0.9% (*v*/*w*)	AWDM, In situ MCT (*L. monocytegenes* contamination of salmon tartare)	[88]
EtOH extract 300 mg/mL	Clinical isolates of *Enterococcus fae-calis*, *E. coli*, *Proteus mirabilis*, *P. aeruginosa*, *S. aureus*, *Staphylococcus lentus*, *Staphylococcus xylosus*, *Streptococcus agalactiae*, *S. pyogenes*	IZ = 21.6 mm (*S. agalactiae*), highest activity IZ = 8.5 mm (*P. aeruginosa)*, lowest activity	AWDM	[148]
Sodium acetate buffer extract (SA) Aqueous extract Chloroform (CHCl_3_)–MeOH extract	*Staphylococcus* sp. isolates (19 strains) causing goat mastitis and *Staphylococcus* sp. isolates (16 strains) causing bovine mastitis	MIC_50_ = 12 μg/mL (bovine mastitis strains) MIC = 100 μg/mL (aqueous extract) MIC_50_ = 3, 6–50 μg/mL (goat mastitis strains) MIC = 25 μg/mL SA and aqueous extracts) MIC > 100 μg/mL (CHCl_3_–MeOH extract)	BMD	[151]
Suspension	*S. aureus* *E. coli* *Candida albicans*	MIC/MBC = 500/500 ppm MIC/MBC = 125/250 ppm MIC/MBC = 62.5/125 ppm IZs = 1.4 mm at 500 ppm 1.6 mm at 1000 ppm), 8.55 mm at 500 ppm and 15.6 mm at 250 ppm	BMD Disc diffusion method (DDM) Agar plate assay	[79]
The dominant hydrolyzed spirulina protein (HSP) Peptide fractions (PF)	*E. coli* *S.* *aureus*	MIC = 625 μg/mL (both HSP and PF) HSP stimulated the bacterial growth PF < 3 kDa inhibited *E. coli* and *S. aureus* growth to 15.2 and 19.6% after 16 h	BMD	[149]
Aqueous extract	*S. aureus*, *P. aeruginosa*, *E. coli*, *Stenotrophomonas maltophiliastrains*	No antibacterial activity	DDM	[145]
MeOH, EtOH, EtOAc, CHCl_3_ extracts	Multidrug-resistant *S. aureus*, *E. coli*, *P. aeruginosa*, *Salmonella* sp. and *Shigella* sp.	Bacterial growth inhibition of 67–98% Most effective extract: MeOH	Kirby-Bauer single-disk diffusion agar method	[150]
Ether, hexano–EtOH, DCM, acetone and MeOH extracts	*S. aureus, B. cereus, B.subtilis*, *E. coli*, *Klebsiella sp, P. aeruginosa*, *S. typhimurium* (ATCC strains) Fungi—*C. albicans* and *Aspergillus brosiliensis*	IZs = 0–43 mm MIC (MeOH extract) = 128 μg/mL against *B. subtilis* was Moderate effect on *C. albicans* Reistance towards *A. brosiliensis*	AWDM DDM	[152]
MeOH extract (100 ng/mL)	*V. parahaemolyticus, V. alginolyticus,* other *Vibrio* sp. *Chromobacterium violaceum*, *S. aureus, P. aeruginosa, E. coli*, *S. marsecens*, *A. hydrophila*	Biofilm inhibition: ~90% (*V. parahaemolyticus)*, 89% (*C. violaceum)*, 88% (*V. alginolyticus*), 74% (*A. hydrophila*), 69–72% (*P. aeruginosa*), 62% (*E. coli*), 61–84% (*S. aureus*), 49% (*S. marsecens*) Extracellular polymeric substance inhibition: 80% (*A. hydrophila*), 62% (*E. coli*), 52–66% (*S. aureus*), less than 30% for the other strains CSH reduction: *A. hydrophila, E. coli* and *S. aureus*	Biofilm inhibition assay—spectrophotometric quantification AWDM BATH assay for CSH determination	[7]
MeOH, acetone, CHCl_3_, hexane extracts	*E. coli*, *S. typhi*, *P. mirabilis*, *V. vulnificus* and *Cellulomonas cellulan*	100 μg MeOH/CHCl_3_ extracts: IZ = 26/27 mm (*E. coli*), IZ = 21 mm (*S. typhi*), IZ = 24 mm (*P. mirabilis*)	AWDM DDM	[146]
EtOH and CHCl_3_ extracts	*Salmonella enterica* serovar *typhi* and *paratyphi*	40 mg/mL EtOH extract IZ = 10–14 mm (*S. paratyphi*), 10–16 mm for (*S. typhi*) CHCl_3_ extract—no inhibitory effect	DDM	[153]
Aqueous, hexane, CHCl_3_, EtOAc and 70% EtOH extracts	*E. coli*, *A. hydrophila*, *S. enterica*, *Klebsiella pneumonie*, *V. cholera Salmonella* Paratyphi, *S. aureus* and *L. monocytogenes* Fungi—*Aspergillus terreus*, *Tirchoderma viride*, *Candida tropicalis* and *S. cerevisiae*	Aqueous extract: no activity Hexane extract IZ = 11 mm (*A. terreus*) CHCl_3_ extract IZ = 21.5 mm (*E. coli*) EtOAc extract: small IZs on *S. paratyphi* and *S. cerevisiae* EtOH extract: moderate activity	DDM	[154]
MeOH extracts, pellet and aqueous extracellular supernatant from *A. platensis*	*B. subtilis*, *E. coli*, *P. fluorescens* Fungi—*C. albicans* and *S. cerevisiae*	MeOH extracts: IZ = 10 mm (*E. coli*), 1.3 mm (*B. subtilis*), IZ >10 mm (*S. cerevisiae*)	AWDM	[155]
Diethyl ether, EtOAc, petroleum ether, n-hexane, CHCl_3_, acetone, MeOH and EtOH extracts Unknown purified antimicrobial compound (PAC)	*E. coli*, *P. aeruginosa*, *B. subtilis*, *B. thuringiensis*, Fungi—*S. cerevisiae*, *Aspergillus flavus* and *A. niger*, *C. albicans*	*E. coli*, *P. aeruginosa*, *B. Subtilis*, *A. niger* were most sensitive Diethyl ether, EtOAc, EtOH extracts inhibited Gram (+) and Gram (-) bacteria; petroleum ether extract inhibited Gram (-); n-hexane extract—no activity MICs (PAC) = 30, 60, 65, 80, 85 µg/mL (*C. albicans*, *B. subtilis*, *S. aureus*, *E. coli* and *P. aeruginosa*) MICs (PAC) > 120 µg/mL (*A. flavus*, *A. niger*)	BMD	[144]
Aqueous and EtOH extract	*S. marcescens*, *E. coli*, *B. cereus*, *M. luteus*, *S. aureus*, *K. pneumoniae* and *P. aeruginosa*, *A. flavus*	EtOH extract: most active on *E. coli* and *S. aureus* *B. cereus* and *K. pneumonie*: most resistant Nitrogen-supplemented medium potentiated the extract’s activity	Agar plate diffusion test	[156]
70% EtOH, 70% MeOH, 70% EtOAc and 70% CHCl_3_ extracts	*S. aureus* isolates	EtOH > MeOH > CHCl_3_ > EtOAc	Microtiter plates reader assay BMD	[157]
Phycocyanin	*S. aureus*, *S. pyogenes*, *B. cereus*, *E. coli*, *P. aeruginosa, S. typhimurium* and *C. albicans*	MIC = 2.1 mg/mL for *C. albicans* and *S. typhimurium*	AWDM BMD	[65]
Dichloromethane (DCM)/MeOH extract, acetone soluble (ASF) and insoluble fraction (AIF) from it, aqueous extract, water insoluble fraction, phycocyanin and culture filtrate	Seven bacterial fish pathogens—*A. hydrophila*, *B. subtilis*, *B. cereus*, *Edwardsiella tarda*, *M. luteus*, *V. parahemolyticus* and *V. alginolyticus*	MICs of ASF = 1.9–15 µg/mL, MBCs (7.8–250 µg/mL) and highest IZs = 31–41 mm. *E. tarda* was the most susceptible pathogen to ASF AIF—moderate effect Phycocyanin IZ on *A. hydrophila* = 16 mm AFM of *A. hydrophila* showed cell wall decay	AWDM BMD Atomic force microscopy (AFM)	[139]
Live cells in a co-culture	Six *Vibrio* bacterial strains—*V. parahaemolyticus*, *V. anguillarum*, V. splendidus, *V. scophthalmi*, *V. alginolyticus* and *V. lentus*	Strong inhibition of bacterial growth ca. 1000 times after 96–120 h co-culturing *V. alginolyticus* and *V. anguillarum*: most resistant slower decrease in bacterial count	Co-culturing of microalgae or cyanobacteria with bacteria—OD measurement	[85]
Intracellular (food-grade solvent) and extracellular EtOH/water (1:1, *v*:*v*) extracts	*S. aureus*, *Salmonella* sp., *E. coli* and *P. aeruginosa*	Slight inhibition of all bacterial species	Mixtures of pathogens and cyanobacteria	[142]
MeOH and acetone extracts (concentration 250–7000 ppm)	*S. aureus* and *S. typhimurium*	IZ (acetone extract at 5000 ppm) = 21.5 mm (*S. aureus*) IZ (MeOH extract) = 17.5 mm (*S. typhimurium*)	AWDM DDM	[143]
EtOH extract	*S. aureus*, *E. coli*, *P. aeruginosa*, *Klebsiella* sp., *Proteus* sp., *Embedobacter* sp.—clinical isolates	IZs = 6–11 mm (11 mm against *E. coli*)	AWDM	[147]
Hexane, EtOAc, EtOH, butanol, acetone, MeOH and CHCl_3_ extracts	*S. aureus*, *E. feacalis*, *S. epidermidis*, *Aeromonas liquefaciens*, *Campylobacter coli*, *Vibrio cholerae*, *Candida glabrata*	IZs butanol extract = 19 mm (*S. epidermidis*), 18 mm (*S. aureus*, *A. liquefaciens*), 13 mm (*C. glabrata*), 12 mm (*E. feacalis*), 11 mm (*C. coli and V. cholera*) and 5 mm (*S. typhi*)	AWDM	[158]
Purified phycocyanin	*S. aureus, Enterococcus durans*, *E. coli*, *K. pneumoniae*, *P. aeruginosa* and *Acinetobacter baumanii*	IZ = 13.3 mm (*E. coli*)*,* 16 mm (*K. pneumoniae*)*,* 18 mm (*P. aeruginosa*), and 9.3 mm (*S. aureus*). MICs = 50–125 µg/mL No activity on *A. baumanii* and *E. durans*	BMD	[159]
EtOH, MeOH and aqueous extracts	Fish and shellfish pathogens—*P. putida*, *P. aeruginosa*, *P. fluorescens*, *A. hydrophila*, *V. alginolyticus*, *V. anguillarum*, *V. fluvialis*, *V. parahaemolyticus, V. harveyi*, *V. fisheri*, *E. tarda* and animal isolates of *E. coli*	EtOH extract: 16 mm IZ and MICs from 100 to 150 µg/mL for *A. hydrophila* EtOH extract: 12 to 15 mm IZs for *Vibrio*, *E. coli* and *E. tarda*	DDM MIC determined by the tube dilution method	[160]
Aqueous, MeOH, EtOH, acetone, petroleum ether and hexane extracts from the EPS or exopolysaccharides	*S. aureus, Staphylococcus epidermidis M. luteus*, *E. coli*, *S. typhimurium* and *P. aeruginosa*	MeOH extract: IZs = 7.5, 11 and 19.5 mm against *M. luteus*, *S. typhimurium* and *P. aeruginosa*, MIC = 1–10 mg/mL, MBC = 10 mg/mL Aqueous extract: IZs = 14 and 7 mm against *S. epidermidis* and *S. typhimurium*, MIC = 5 mg/mL and MBC = 12 mg/mL Neither extract was active against *E. coli* and *S. aureus*	DDM BMD	[161]
Phenolic compounds (PC), 1.15 mg/g biomass from a MeOH extract	*A. flavus*	PC decreases the dry mass of *A. flavus*—1.6, 1.2 and 1.3 times (regarding glucosamine, ergosterol and protein content) Up to 56% inhibition of glucosamine	Protein content (micro-Kjeldahl method, dry weight) and ergosterol content in the mycelium	[141]
Hexane, CHCl_3_, EtOAc, acetone and MeOH extracts	*B. subtilis*, *B. cereus*, *Enterobacter aerogenes*, *E. coli*, *K. pneumoniae*, *L. monocytogenes*, *M. luteus*, *P. mirabilis*, *P. aeruginosa*, *S. typhi*, *S. aureus*, *E. faecalis* and *Y. enterocolitica*	MIC = 200–500 ppm Hexane extract IZs = 9–11 mm, acetone extract IZs = 10–12 mm, MeOH extract IZs = 11–16 mm, EtOAc IZs = 11–17 mm, highest for *P. mirabilis*, CHCl_3_ IZs = 18 mm *B. subtilis* (MIC at 200 ppm) and *M. luteus*	AWDM BMD	[68]
Proteinic, hydroethanol (hydroEtOH) and tannic extract and fraction of terpene–sterols	*S. aureus, E. coli, S. typhi, Salmonella B, S. flexneri*, *C. albicans*	All extracts inhibit *S. flexneri*, *C. albicans* Proteinic, hydroEtOH and tannic extract—*S. typhi* Iroteinic and hydroEtOH extracts—*Salmonella B* HydroEtOH extract*—E. coli* Tannic extract—*S. aureus*	-	[162]
EtOH, n-butanol, CHCl_3_ and water extract	*M. tuberculosis*	No satisfactory results	Absolute concentration method	[163]
Aqueous, EtOH and MeOH extracts	*P. putida*, *P. fluorescens*, *P. aeruginosa*, *A. hydrophila* (different strains), *V. algi-nolyticus*, *V. parahaemolyticus*, *V. harveyi*, *V. fluvialis*, *V. fisheri*, *V. anguil-larum*, *E. coli* (different strains) and *E. tarda*	EtOH extracts: no activity against *E. coli* strains O111 and O109, IZ = 10 mm (*A. hydrophila* AH1), IZ—15.6 mm (AH2 and *E. coli* O1 and O115). IZs = 8.3 to 10.3 mm (*A. hydrophila* AH3).	Single DDM	[164]
EtOH, MeOH and acetone (1:1:1) extract (21 days maceration)	Fish pathogens	The IZ = 17, 16 and 15 mm (*Proteus mirabilis*, *Bacillus pumilus* and *Mammaliicoccus sciuri*), IZ of antibiotics = 16–26, 16–18 or 12–20 mm	AWDM	[165]

**Table 3:** A review of the in vitro research on the antimicrobial activity of *Arthrospira*. spp., including unidentified ones.

Species	Extract or Sample	Test Microorganism	Antibacterial Activity—MIC, MBC, IZ, etc.	Test Methods	Reference
*Spirulina* sp.	35% Polyhydroxybutyrate (PHB) nanofibers with 1% phenolic compounds both from *Spirulina* LEB 18	*S. aureus* and *E. coli*	IZ = 7.5 mm (*S. aureus*) *E. coli* with higher resistance The phenolic compounds did not lose their activity after the electrospinning This new nanomaterial combines a biodegradable food packaging function with an antibacterial activity	DDM	[91]
*Spirulina* sp.	MeOH and EtOH extracts	*S. aureus*, *B. cereus*, *P. aerugenosa*, *E. col*, and *S. typhimurium*; fungi—*C. albicans*, *Fusarium graminearum*, *Fusarium moniliforme*, *Aspergillus ochraceus*, *Penicillium verrucosum* and *Malassezia pachydermatis*	The IZs of the EtOH extract against were in the range of 7–10 mm, except for *F. graminearum* and *A. ochraceus* (0 mm). The MeOH extract had no activity for the bacteria and *C. albicans* and IZs against the rest were in the range of 7.5–9 mm	AWDM	[166]
Two strains of *A. maxima*	MeOH extract, pellet, aqueous extracellular supernatant	*B. subtilis*, *E. coli*, *P. fluorescens*, *C. albicans* and *S. cerevisiae*.	The MeOH extract of one strain had IZ ˂ 10 mm against *S. cerevisiae*	AWDM	[155]
*A. maxima*	Hemolymph of the abalone *Haliotis laevigata* fed with *A. maxima*	*Vibrio anguillarum*	No antibacterial activity	MTS assay	[167]
*A. maxima*	Aqueous and MeOH extracts	*E. coli, P. vulgaris*, *S. aureus*, *B. subtilis* and *C. albicans*	MeOH extract IZs = 36.5–44.8 mm, except *C. albicans* (smaller IZ)*,* not a significant difference at 0.5 and at 0.66 g/mL Aqueous extract IZ ≥ 50 mm (*S. aureus*) at 0.15 and 0.2 g/mL but at 0.33 g/mL no effect *B. subtilis:* effect only at 0.33 g/mL (13.5 mm) The IZs for the other species = 11.5 to 14 mm	DDM	[168]
	Pure phycocyanin	*B. cereus*, *B. subtilis*, *S. aureus*, *M. luteus*, *S. marcescens* and *K. pneumoniae*	Four mg per disk: IZs 18–20 mm MIC = 300 µg/mL for all Total phycobiliprotein content and phycocyanin increased under salt stress	DDM	[169]

## 7. Antimicrobial Activity of *Dunaliella* spp.

Substantial in vitro research, as well as some in vivo studies, has been conducted in regard to *D. salina* and other *Dunaliella* spp. (Table 4 and Table 5).

To the best of our knowledge, two in vivo studies have been carried out studying *D. salina*. Research was carried out onto plant pathogens, in addition to in vitro tests. A hexane extract from the algal species had an MIC of only 0.3 mg/mL against *B. subtilis* and 3 mg/mL against the other bacteria. The other two extracts had MICs of 3 mg/mL on *B. subtilis* and >3 mg/mL on the other bacteria. *Pseudomonas syringae* was the most resistant in the DDM, with inhibition zones (IZs) 8–12 mm (24 mm for ciprofloxacin), while *B. subtilis* was the most sensitive, again with IZs of 20 and 21 mm of the hexane and EtOH extracts, respectively (32 mm for the control). The mean IZ of the extracts on *Pectobacterium carotovorum* was half that of ciprofloxacin (20 mm). The hexane extract showed the highest amount of carotene, so it was selected for in vivo analyses. The leaves and fruits of tomato plants and zucchini fruits were artificially wounded, and treated groups were sprayed with or immersed in a hexane extract. Then, the respective bacterial suspension was (spray)-inoculated. The tomato plants that were treated showed a reduction in bacterial speck spot disease of 66% in incidence and 77% in severity. Similarly, treated tomato and zucchini fruits showed less signs of soft rot, with disease incidences of 5% and 13% compared to 91% and 100%, respectively, for the positive control [170].

Another in vivo research studied the effect of chitosan nanoparticles loaded with hexane: ethyl acetate (HEAE-CNPs) and methanol (ME-CNPs) extracts on wound healing. As noted, antioxidants such as carotenoids contribute to wound healing, as they are scavengers of ROS and anti-inflammatory agents, e.g., NF-κβ signaling pathways inhibitors. The hexane extract contained 19 mg/g β-carotene and 16 mg/g zeaxanthin, while the MeOH extract contained only traces of zeaxanthin. That would explain the better effect of the hexane extract. Both agents increased vascular endothelial growth factor, angiogenesis, collagen skin contents and tissue remodeling and decreased (TNF)-α. Therefore, both HEAE-CNPs and ME-CNPs exerted wound healing and regeneration not only due to the antibacterial effect but also to the antioxidant and anti-inflammatory activity [171].

Another in vivo research deployed intragastrical inoculation of mice with *H. pylori* three times at two-day intervals. Two weeks after inoculation, mice were treated orally with the total carotenoid extract of a *Dunaliella* sp. (100 mg/kg body weight per day, 0.15–0.18 mL in corn oil) for two weeks, during which stomach and spleen were removed and prepared for further histopathology analysis. The histological changes of the infection started with the denudation of the surface epithelium that affected the gastric pit, followed by gastric gland hyperplasia and lamina propria swelling. After 14 days of treatment, the *Dunaliella* sp. treatment group showed no hyperplasia gastric gland, reduced swelling of lamina propria and normal gastric pit condition. Unlike *Chlorella* sp., *Dunaliella* sp. did not delay the gastric ailment but promoted the healing process during gastritis and restored the normal histology of the stomach relatively rapidly, e.g., faster than *Isochrysis* sp. It is known that β-carotene reduces the inducible nitric oxide synthases and cyclooxygenase-2 expression and suppresses the ROS inflammation and inflammatory signaling during gastritis, thus accelerating the gastric healing [172]. In addition, a high intake of carotenoids such as astaxanthin has been suggested to prevent gastritis by lowering colonization levels of *H. pylori*. The total carotenoid present in dry weight of *Dunaliella* sp. was especially high (99%). Since *Dunaliella* sp. treatment healed the stomach in 14 days, authors supported the view that carotenoids, which combine antibacterial and antioxidant effect, may be a new strategy for treating gastritis-causing *H. pylori* [173].

Previous studies have found oleic, alpha-linoleic and palmitic acids to be the major components in the EtOH or hexanic extract of *D. salina* and to be mainly responsible for its in vitro antimicrobial activity. The EtOH extract also contained the highest amount of EtOH-extractable PUFAs (77%), of which linolenic acid was 45% [58]. There is a hypothesis that free fatty acids and phytol are derived from galactolipid and chlorophyll catabolism in that microalgae (but likely not limited to *D. salina*). Together with the products related to carotenoid degradation, they act as antimicrobials but can likely be extracted only by a sufficiently aggressive extraction protocol. Probably for that reason, EtOAc and MeOH extracts from a hexane extract lacked antimicrobial activity against *E. coli* and *S. aureus* in the study of Iglesias et al., 2019 [174]. Another study concluded that not only the several fatty acids but also the polysaccharide-rich fraction and the compounds cyclocitral, neophytadiene and phytol explained the antimicrobial properties of *D. salina* [3,175]. Pressurized extracts from the same species also contained β-cyclocitral, α and β-ionone, neophytadiene, phytol and hexadecanoic acid. They are compounds with documented antimicrobial activity and were present in higher amounts in the most active antibacterial extracts. The major compound in the pressurized extracts was identified as 9,12,15-octadecatrienoic acid methyl ester, and all extracts yielded fifteen different volatile compounds as a whole, as well as several fatty acids (mainly palmitic, α-linolenic and oleic acids) that could also have been responsible for the antimicrobial activity were identified in the extracts [83].

Other unique compounds which are potential antimicrobial agents, such as 3,3,5-trimethylheptane and n-hexadecane, have also been revealed [175]. Maadane et al., 2017, found that the antimicrobial effect of *D. salina* and other *Dunaliella* species correlated with the content of fatty acids, which were main compounds of the biomass, but also with carotenoids and polyphenols. The authors suggested additive or synergetic action for them [61,140].

Biofouling or biological fouling is defined as the accumulation of microorganisms, plants, filamentous algae or small invertebrate animals on ships or other surfaces where it is not wanted, such in as submarine hulls and devices such as water inlets, pipework, ponds and rivers, where it degrades the item’s main purpose [176]. To date, the testing of the anti-fouling properties of *D. salina* and other species from the genus turned out to be negative [177].

According to previous studies, among extract from different microalgal species, only the EtOH extracts from *Dunaliella* sp. contained a significant quantity of carotenoids; therefore, they could also be responsible for the antibacterial effect [61]. The antimicrobial activity of *Dunaliella primolecta* is likely due to pheophorbide *α* and *β*-like compounds [3], while that of *Dunaliella tertiolecta*—to polyphenols, notably gentisic acid, (+) catechin and (−) epicatechin [178].

The same species (*D. tertiolecta*) had no clear anti-*Vibrio* activity when used in vivo as green-water cultures in *Vibrio*-challenged *L. vannamei* cultures. That study is interesting, however, because no differences in mortality of juvenile *L. vannamei* were observed between different groups of treatments and the control, suggesting that the pathogenicity of *V. campbellii* could be related to the growth stage of shrimps [84].

**Table 4:** A review of the research on the antimicrobial activity of *Dunaliella salina* (Dunal) Teodoresco.

Extract or Sample	Test Microorganism	Antimicrobial Activity—MIC, MBC, IZ, etc.	Test Methods	Reference
EtOH extract from *D. salina* from Moroccan coastlines	*S. aureus*, *E. coli* and *P. aeruginosa*; *C. albicans* and *A. niger*	MIC = 3.4 mg/mL (*E. coli*), 2.6 mg/mL (*P. aeruginosa*), >5 mg/mL (*C. albicans*) No effect against *S. aureus* and *A. niger* at 5 mg/mL	BMD	[60,140]
Pressurized liquid extracts (hexane, petroleum ether, hexane and water) and extraction conditions of 40, 100 and 160 °C.	*S. aureus*, *E. coli* *C. albicans* and *A. niger*	Best activity for each solvent at 160 °C Petroleum ether and hexane—lowest MBCs (6 mg/mL for *E. coli*), *S. aureus*: less susceptible, followed by *C. albicans* and *A. niger*. Lowest MFC: *A. niger*—32 mg/mL	BMD Agar plate assay	[83]
EtOAc and MeOH extracts from a hexane extract	Clinical biofilm-forming bacterial strains: *S. aureus*, coagulase-negative *Streptococcus* (CNS), *S. epidermidis*, *E. coli*, *K. pneumonie, E. cloacae* and *P. aeruginosa* Fungi: (*C. albicans* and *Candida parapsilosis* resistant to fluconasole)	No antibacterial and antibiofilm effect on the test strains (different resistance profiles towards clinical treatments, e.g., *S. aureus*, *E. coli* and *C. parapsilosis*)	BMD Biofilm formation assay	[174]
CHCl_3_/MeOH/acetone (in ratio of 2/1/1) extract	*S. mutans* involved in dental plaque and consequent dental cavities formation	Best IZs at the highest doses: 18.5 (6 mg/disc) and 25.4 mm (7.5 mg/mL) in the AWDM. Antibiofilm activity at 2 mg/mL	DDM AWDM	[179]
EtOH extracts from *D. salina* from the Aegean Sea	Pathogenic bacteria: *E. faecalis, S. aureus* and MRSA, *E. coli*, *K. pneumoniae*, *S. flexneri*, *V. cholerae* Fungi: *A. fumigatus*, *C. albicans* and *C. neoformans* Other bacteria: *A. hydrophila, A. salmonicida, A. borkumensis, Alcanivorax* sp., *Allivibrio salmonicida*, *E. litoralis, P. donghaensis*, *Pseudoalteromonas* sp., *P. mendocina* and *V. furnissii*	No effect	AWDM (1 mg/disc) BMD	[177]
N-hexane, DCM, EtOH and MeOH extracts and fatty acid oil	Fish and clinical/foodborne pathogens: *Yersinia ruckeri*, *Lactococcus garvieae*, *Vibrio anguillarum*, and *V. alginolyticus*, *Y. enterocolitica*, *S. aureus*, *L. monocytogenes*, *M. luteus*, *B. cereus*, *E. coli*, *S. enteritidis*, *P. aeruginosa*, *Shigella sonnei*, *B. subtilis* and *C. albicans*	Maximal IZ (EtOH extract) = 22.9 mm (*Y. enterocolitica*) MeOH and EtOH extracts: most active MeOH extract: not effective on *B. subtilis* and *S. enteritidis*—MBC = 0.32–10 mg/mL EtOH extract: MBC = 0.63 mg/mL on fish pathogens	DDM, BMD	[58]
Extracts made through different solvents and solvent mixtures (1:1)	Selected human pathogens: *V. cholerae*, *K. pneumoniae*, *E. coli*, *P. aeruginosa*, *Salmonella* sp., *Proteus* sp., *Streptococcus pyogens*, *S. aureus*, *B. megaterium* and *B. subtilis*	Highest IZs = 10 mm: CHCl_3_:MeOH crude extract (*S. aureus*, *S. pyogenes* and *V. cholerae*), Gram (−) bacteria > Gram (+) Acetone: CHCl_3_ extract (*S. pyogens*) Minimum IZ = 2 mm (isopropanol extract on *Proteus* sp.)	DDM	[175]
Hemolymph of the abalone *Haliotis laevigata* fed with *D. salina*	*V. anguillarum*	No antibacterial activity	MTS assay	[167]
EtOH, n-butanol, CHCl_3_ and water extracts	*M. tuberculosis*	No satisfactory results	Absolute concentration method	[163]
CHCl_3_/MeOH, EtOH and hexane extracts with concentrations of 350, 214 and 97 mg/mL, respectively, for in vitro test; hexane extract at 5 or 10 g/L for in vivo tests	*Pseudomonas syringae* pv. *tomato* (causing bacterial speck spot), *Pectobacterium carotovorum* subsp*. carotovorum* (causing soft rot), *B. subtilis*; the first two bacteria used also for in vivo studies with hexane extracts on young tomato plants and fruits of tomato and zucchini, respectively	Hexane extract: MIC = 0.3 mg/mL/IZs = 20 mm (*B. subtilis*), MIC = 3 mg/mL (the rest) EtOH extract: IZ = 21 mm (*B. subtilis*) Treated tomato plants: a reduction in bacterial speck spot disease (66% in incidence, 77% in severity) Treated tomato and zucchini fruits: less signs of soft rot, 5% and 13% disease incidences	BMD DDM In vivo application to bacterial speck spot and soft rot on plant tissues	[170]
Shrimps fed with *D. salina*	45 shrimps infected by *V. alginolyticus* and V*. harveyi* reared for 21 days in vivo	A decrease in bacteria amount, algae have a potential to be used as biocontrols	Total plate count method	[180]
Chitosan nanoparticles loaded with extracts—hexane:ethyl acetate (HEAE-CNPs) and methanol (ME-CNPs) gels	Wistar rats	Topical applications of HEAE-CNP and ME-CNP gel (10 mg/kg): wound healing by 5-fold and 3.6-fold, after 10 days, respectively	In vivo test: 5 mm excision wounds made by a punch needle	[171]

**Table 5:** A list of the research on the antimicrobial activity of different Dunaliella spp., including unidentified ones.

Species	Extract or Sample	Test Microorganism	Antimicrobial Activity—MIC, MBC, IZ, etc.	Test Methods	Reference
*Dunaliella* sp.	EtOAc and MeOH extracts from a hexane extract	Biofilm-forming strains causing clinical infections: *S. aureus*, CNS, *S. epidermidis*, *E. coli*, *K. pneumonia*, *E. cloacae* and *P. aeruginosa*; fungi (*C. albicans* and *C. parapsilosis* resistant to fluconasole)	No antibacterial and biofilm-inhibiting effect activity on the test strains, which included strains with different resistance profiles towards clinical treatments, such as *S. aureus*, *E. coli* and *C. parapsilosis*	BMD, biofilm formation assay	[174]
*Dunaliella* sp. from Moroccan coastlines	EtOH extract	*E. coli*, *S. aureus* and *P. aeruginosa*; fungi—*C. albicans* and *A. niger*	Moderately inhibited *E. coli*, *S. aureus* and *C. albicans* with MIC > 5 mg/mL, and *P. aeruginosa* with MIC 4.3 mg/mL but not *A. niger* at 5 mg/mL	BMD	[61]
*Dunaliela* sp. from the Aegean Sea	EtOH extracts	Ten pathogens—*E. faecalis*, *S. aureus* and MRSA; *E. coli*, *K. pneumoniae*, *S. flexneri* and *V. cholera*; fungi—*A. fumigatus*, *C. albicans* and *C. neoformans*; the fouling bacteria *A. hydrophila*, *A. salmonicida*, *A. borkumensis*, *Alcanivorax* sp., *Allivibrio salmonicida*, *E. litoralis*, *P. donghaensis*, *Pseudoalteromonas* sp., *P. mendocina* and *V. furnissii*	No effect	AWDM (1 mg/disc), BMD	[177]
*D. tertiolecta*	Aqueous, sodium acetate and CHCl_3_-MeOH extracts	*Staphylococcus* spp. isolates causing goat (19 strains) and bovine (16 strains) mastitis	MIC_50_ = 3–25 μg/mL, MICs = 25 μg/mL (aqueous extract), >100 μg/mL (the rest against bovine mastitis strains) MICs = 50, 100 and >100 μg/mL μg/mL (goat mastitis strains)	BMD	[151]
*D. tertiolecta*	MeOH extract	114 bacterial and 11 fungal strains from ear swabs from patients with external otitis using for over one year: *Staphylococcus* spp. (28.8%) and *P. aeruginosa* (24.8%). Many of the strains, except *Klebsiella* spp., could form biofilms. Only three *S. aureus* strains and 11 CNS showed resistance to methicillin	MIC_50_ and the MIC ranges against *S. aureus* = 5.6 × 10^9^ and 2.8 × 10^9^–1.1 × 10^10^ algae cells/mL, *P. aeruginosa* = 2.8 × 10^9^ and 1.4 × 10^9^–5.6 × 10^9^ algae cells/mL, Enterobacteriaceae (strains of *E. coli* and *Klebsiella* spp.,) = 2.2 × 10^10^ and 1.1 × 10^10^–2.2 × 10^10^ algae cells/mL	BMD (MIC measured as algae cells/mL)	[178]
*D. tertiolecta*	Hexane extract and DCM and MeOH fractions from it	*E. coli*, *P. aeruginosa*, *Klebsiella pneumoniae*, *B. subtilis*, *S. aureus* and *M. luteus*	Active only against *B. subtilis* and *S. aureus* with IZs ranging from 8.9 to 11.6 mm	Agar diffusion method (the Oxford cup method)	[181]
*D. parva*	dH_2_O extracts made with freezing and thawing	Four test strains of opportunistic bacteria (*E. coli, K. ozaenae, P. aeruginosa* and *S. aureus*)	<20% growth inhibition; peloid-containing extracts of cells had a pronounced antibacterial effect against opportunistic bacteria	BMD, photometric method	[56]
*Dunaliella* sp.	Total carotenoid extract	*E. coli*, *Salmonella* sp., *P. aeruginosa*, *B. cereus*, *Klebsiella* sp.; Mice inoculated with *H. pylori*	*E. coli* and *Salmonella* sp.: no activity up to 100 mg/mL; *P. aeruginosa*: probiotic activity at 100 mg/mL only; *B. cereus*: inhibition at 6.25 mg/mL; *Klebsiella* sp.: inhibition at 25 mg/mL. From three microalgal species, only *Dunaliella* sp. healed the stomach in 14 days, highest ability to promote gastric healing due to antioxidant and antimicrobial effect	DDM, in vivo gastritis studies on model mice	[173]
*D. tertiolecta*	Aqueous and acidified MeOH extract	*V. campbellii*, *Vibrio*-challenged *L. vannamei* shrimp cultures	12–13% growth inhibition (the aqueous extract at 78–313 μg/mL. Exact MIC not determined due to variation in the inhibition at different concentrations. No clear anti-*Vibrio* activity for the MeOH extract and in the in vivo test when the alga was used as green-water cultures	BMD, in vivo shrimp challenge assay	[84]
*D. tertiolecta*	Biomass as a feed	*Artemia* (brine shrimp) challenged with *Vibrio*	Full protection against vibriosis in terms of survival and viable parameters. This effect could be due to the immune-stimulating effect or antibacterial compounds	In vivo challenge test after daily feeding	[122]

## 8. Evaluation of the In Vitro Antimicrobial Activity of *Arthrospira* and *Dunaliella* According to Different Criteria

We assessed the antimicrobial activity of the two genera according to different criteria. Different standards apply when evaluating antimicrobial or antibacterial activity in vitro. Today, we still use Eloff’s criteria, which state that an extract or fraction has significant antibacterial activity if its MIC against a particular microorganism is equal to or lower than 0.1 mg/mL [182]. Plant extracts with MIC values up to 0.5 mg/mL are regarded as strong inhibitors, those between 0.6 and 1.5 mg/mL as moderate inhibitors and those above 1.6 mg/mL as weak inhibitors, according to Aligiannis et al., 2001 [183].

Based on the diameter of the IZ, antimicrobial activity can be divided into four classes, according to Greenwood (1995). When an IZ is greater than 20 mm, the antimicrobial effect is strong; at 16–20 mm, it is medium; at 11–15 mm, it is weak; at <10 mm, it is nonexistent [184]. Other authors claim that an IZ with a diameter of more than 15 mm exhibits strong inhibitory activity, a diameter of 9–14 mm exhibits moderate inhibitory activity and a diameter of less than 8 mm exhibits weak inhibitory activity [185,186].

The antimicrobial activity of *A. platensis* is strain-specific and also varies with the extraction solvent and procedure. The antimicrobial properties vary from no or weak activity through moderate to strong and excellent according to all criteria and sometimes are comparable or exceed the activity of the control antibiotics.

The most potent ingredient, arguably, is an acetone soluble fraction from a strain with MICs as low as 1.9–15 µg/mL and IZs as high as 31–41 mm against *E. tarda* and other bacterial fish pathogens including *Bacillus* and *Vibrio* spp. [139]. The sodium acetate and aqueous extracts of *A. platensis* had MIC values of 25 μg/mL against *Staphylococcus* sp. [151]. The MeOH and hexano-EtOH extracts of another strain inhibited *B. subtilis* and *B. cereus* with IZs of 43 and 41 mm, respectively. The latter had an IZ of 28 mm on *E. coli,* and together with the IZ of the acetone extract of 24 mm, they exceeded the effect of gentamycin (23 mm) [152].

Other *Arthrospira* species generally had weak to moderate and rarely strong effect, except for *A. maxima*, whose aqueous extract inhibited *S. aureus* with more than 50 mm IZ, while the MeOH extract showed IZs of 37–45 mm against *E. coli*, *P. vulgaris*, *S. aureus* and *B. subtilis* [168].

Purified compounds generally have higher activity than extracts; therefore, the strong activity of purified phycocyanin, which had MIC values against *E. coli*, *K. pneumoniae* and *P. aeruginosa* of 100, 75 and 50 µg/mL, respectively, was expected [159]. Also anticipated was the significant effect of a purified unidentified compound with MICs in the range of 30–85 µg/mL against *C. albicans*, *B. subtilis*, *S. aureus*, *E. coli* and *P. aeruginosa* [144].

The activity of *D. salina* is also strain-specific and varies from no effect to strong inhibition. The CHCl_3_/MeOH/acetone extract had an IZ of 25.4 mm against caries-causing *S. mutans* [179]. An EtOH extract had an IZ of 23 mm against *Y. enterocolitica* and an MBC of 0.63 mg/mL against fish bacterial pathogens. A DCM extract from the same strain had an MBC value of 0.32 mg/mL against *B. subtilis*. MICs are at lower concentrations than MBCs, meaning this strain fulfills the criteria of Aligiannis and most likely of Eloff for significant activity [58]. Hexane and EtOH extracts against *B. subtilis* had IZs of 20 and 21 mm, respectively and an MIC of 0.3 mg/mL for the hexane extract [170]. Other *Dunaliella* species generally had weak or no effect, except for *D. tertiolecta,* with MICs as low as 25, 50 and 100 μg/mL for the aqueous and sodium acetate extract against some *Staphylococcus* spp. [151].

## 9. Conclusions

In addition to their already exploited roles for nutraceuticals, and in biotechnology, the genera *Arthrospira* and *Dunaliella* have different bioactivities and potential in medical applications due to their high diversity of bioactive compounds. One such activity is the antimicrobial action (antibacterial and antifungal), which varies in a broad range for the two genera and is strain-specific. There are strains of *A. platensis* with very potent activity with MIC values as low as 1.9–15 µg/mL, and *Arthrospira* sp. with an IZ of 50 mm. Noteworthy are the substantial amounts of in vivo studies of *Arthrospira* which show that the cyanobacterium is very promising for preventing vibriosis in shrimp and *Helicobacter pylori* infection and for wound healing. The innovative laser irradiation of the chlorophyll it releases can cause photodynamic destruction of bacteria. *D. salina* exhibits MIC values lower than 300 µg/mL and an IZ value of 25.4 mm, while *D. tertiolecta* has demonstrated MIC values of 25 and 50 μg/mL. These values fulfill all criteria for significant antimicrobial activity and sometimes are comparable or exceed the activity of the control antibiotics. The antimicrobial bioactive compounds which are responsible for that action are fatty acids including PUFAs, polysaccharides, glycosides, peptides, neophytadiene, etc. Cyanobacteria, such as *Arthrospira*, also particularly have antimicrobial flavonoids, terpenes, alkaloids, saponins, quinones and some unique-to-them compounds, such as phycobiliproteins, polyhydroxybutyrate, the peptide microcystin, etc. All these substances are subject to maximization by stress factors in a two-step process during controlled fermentation conditions.

The current state of the art shows that the antimicrobial metabolites from *Arthrospira* and *Dunaliella* hold potential, but the market products are still limited, which is valid for other microalgal species too. More research is warranted so that they can reach practical application.

## Data Availability

Research data can be obtained from the authors by e-mail.

## References

[1] Adhoni S.A., Thimmappa S.C., Kaliwal B.B. (2016). Phytochemical Analysis and Antimicrobial Activity of Chorella Vulgaris Isolated from Unkal Lake. J. Coast. Life Med..

[2] Habibi Z., Imanpour Namin J., Ramezanpour Z. (2018). Evaluation of Antimicrobial Activities of Microalgae Scenedesmus Dimorphus Extracts against Bacterial Strains. Casp. J. Environ. Sci..

[3] Saha S.K., Mchugh E., Murray P., Walsh D.J. (2015). Microalgae as a Source of Nutraceuticals (Book Chapter). Phycotoxins: Chemistry and Biochemistry.

[4] Pina-Pérez M.C., Rivas A., Martínez A., Rodrigo D. (2017). Antimicrobial Potential of Macro and Microalgae against Pathogenic and Spoilage Microorganisms in Food. Food Chem..

[5] Amaro H.M., Guedes A., Malcata F., Mendez-Vilas A. (2011). Antimicrobial Activities of Microalgae: An Invited Review. Science against Microbial Pathogens: Communicating Current Research and Technological Advances.

[6] Antibiotics from Nature: Traditional Medicine as a Source of New Solutions for Combating Antimicrobial Resistance|AMR Control. http://resistancecontrol.info/rd-innovation/antibiotics-from-nature-traditional-medicine-as-a-source-of-new-solutions-for-combating-antimicrobial-resistance/.

[7] LewisOscar F., Nithya C., Bakkiyaraj D., Arunkumar M., Alharbi N.S., Thajuddin N. (2017). Biofilm Inhibitory Effect of Spirulina Platensis Extracts on Bacteria of Clinical Significance. Proc. Natl. Acad. Sci. India Sect. B —Biol. Sci..

[8] Gheda S.F., Khalil M.A., Gheida S.F. (2013). In Vitro and In Vivo Preliminary Results on Spirulina Platensis for Treatment of Impetigo: Topical Cream Application. African J. Biotechnol..

[9] Rasoanaivo P., Wright C.W., Willcox M.L., Gilbert B. (2011). Whole Plant Extracts versus Single Compounds for the Treatment of Malaria: Synergy and Positive Interactions. Malar. J..

[10] Wright C.W., Linley P.A., Brun R., Wittlin S., Hsu E. (2010). Ancient Chinese Methods Are Remarkably Effective for the Preparation of Artemisinin-Rich Extracts of Qing Hao with Potent Antimalarial Activity. Molecules.

[11] Elfawal M.A., Towler M.J., Reich N.G., Weathers P.J., Rich S.M. (2015). Dried Whole-Plant Artemisia Annua Slows Evolution of Malaria Drug Resistance and Overcomes Resistance to Artemisinin. Proc. Natl. Acad. Sci. USA.

[12] Iwu M.M., Duncan A.R., Okunji C.O. (1999). New Antimicrobials of Plant Origin. Perspect. New Crop. New Uses.

[13] Parimelazhagan T. (2016). Pharmacological Assays of Plant-Based Natural Products.

[14] Kumarasamy Y., Cox P.J., Jaspars M., Nahar L., Sarker S.D. (2002). Screening Seeds of Scottish Plants for Antibacterial Activity. J. Ethnopharmacol..

[15] Sánchez J., Curt M.D., Robert N., Fernández J. (2019). Capter Two-Biomass Resources. The Role of Bioenergy in the Bioeconomy: Resources, Technologies, Sustainability and Policy.

[16] Miller L.H., Su X. (2011). Artemisinin: Discovery from the Chinese Herbal Garden. Cell.

[17] Fairhurst R.M., Nayyar G.M.L., Breman J.G., Hallett R., Vennerstrom J.L., Duong S., Ringwald P., Wellems T.E., Plowe C.V., Dondorp A.M. (2012). Artemisinin-Resistant Malaria: Research Challenges, Opportunities, and Public Health Implications. Am. J. Trop. Med. Hyg..

[18] Pereira L. (2021). Macroalgae. Encyclopedia.

[19] Turland N.J., Wiersema J.H., Barrie F.R., Greuter W., Hawksworth D.L., Herendeen P.S., Knapp S., Kusber W.-H., Li D.-Z., Marhold K. (2018). International Code of Nomenclature for Algae, Fungi, and Plants (Shenzhen Code) Adopted by the Nineteenth International Botanical Congress Shenzhen, China, July 2017, Regnum Vegetabile 159.

[20] Wehr J.D. (2015). Freshwater Algae of North America: Ecology and Classification.

[21] Raven J.A., Giordano M. (2014). Algae. Curr. Biol..

[22] Thoré E.S.J., Muylaert K., Bertram M.G., Brodin T. (2023). Microalgae. Curr. Biol..

[23] Sathasivam R., Radhakrishnan R., Hashem A., Abd_Allah E.F. (2019). Microalgae Metabolites: A Rich Source for Food and Medicine. Saudi J. Biol. Sci..

[24] Plaza M., Santoyo S., Jaime L., Avalo B., Cifuentes A., Reglero G., García-Blairsy Reina G., Señoráns F.J., Ibáñez E. (2012). Comprehensive Characterization of the Functional Activities of Pressurized Liquid and Ultrasound-Assisted Extracts from Chlorella Vulgaris. LWT—Food Sci. Technol..

[25] Jacob J.P., Mathew S. (2017). Effect of Lipases from Candida Cylinderacea on Enrichment of PUFA in Marine Microalgae. J. Food Process. Preserv..

[26] Kiuru P., Valeria D’Auria M., Muller C.D., Tammela P., Vuorela H., Yli-Kauhaluoma J. (2014). Exploring Marine Resources for Bioactive Compounds. Planta Med..

[27] Bays H.E., Tighe A.P., Sadovsky R., Davidson M.H. (2008). Prescription Omega-3 Fatty Acids and Their Lipid Effects: Physiologic Mechanisms of Action and Clinical Implications. Expert Rev. Cardiovasc. Ther..

[28] Kannan N., Rao A.S., Nair A. (2021). Microbial Production of Omega-3 Fatty Acids: An Overview. J. Appl. Microbiol..

[29] Younes A., Yasothan U., Kirkpatrick P. (2012). Brentuximab Vedotin. Nat. Rev. Drug Discov..

[30] Gentscheva G., Nikolova K., Panayotova V., Peycheva K., Makedonski L., Slavov P., Radusheva P., Petrova P., Yotkovska I. (2023). Application of Arthrospira Platensis for Medicinal Purposes and the Food Industry: A Review of the Literature. Life.

[31] Chamorro G., Salazar M., de Lima Araújo K.G., dos Santos C.P., Ceballos G., Castillo L.F. (2002). Actualización En La Farmacología de Spirulina (Arthrospira), Un Alimento No Conven-Cional [Update on the Pharmacology of Spirulina (Arthrospira), an Unconventional Food]. Arch Latinoam Nutr..

[32] Hoseini S.M., Khosravi-Darani K., Mozafari M.R. (2013). Nutritional and Medical Applications of Spirulina Microalgae. Mini Rev. Med. Chem..

[33] Shao W., Ebaid R., El-Sheekh M., Abomohra A., Eladel H. (2019). Pharmaceutical Applications and Consequent Environmental Impacts of <em>Spirulina (Arthrospira)</Em>: An Overview. Grasas Aceites.

[34] Shalaby E. (2011). Algae as Promising Organisms for Environment and Health. Plant sSgnaling Behav..

[35] Oren A. (2005). A Hundred Years of Dunaliella Research: 1905–2005. Saline Systems.

[36] Mobin S., Alam F. (2017). Some Promising Microalgal Species for Commercial Applications: A Review. Energy Procedia.

[37] Montero-Lobato Z., Vázquez M., Navarro F., Fuentes J.L., Bermejo E., Garbayo I., Vílchez C., Cuaresma M. (2018). Chemically-Induced Production of Anti-Inflammatory Molecules in Microalgae. Mar. Drugs.

[38] Falaise C., François C., Travers M.A., Morga B., Haure J., Tremblay R., Turcotte F., Pasetto P., Gastineau R., Hardivillier Y. (2016). Antimicrobial Compounds from Eukaryotic Microalgae against Human Pathogens and Diseases in Aquaculture. Mar. Drugs.

[39] Senhorinho G.N.A., Ross G.M., Scott J.A. (2015). Cyanobacteria and Eukaryotic Microalgae as Potential Sources of Antibiotics. Phycologia.

[40] Bhalamurugan G.L., Valerie O., Mark L. (2018). Valuable Bioproducts Obtained from Microalgal Biomass and Their Commercial Applications: A Review. Environ. Eng. Res..

[41] Cai J., Lovatelli A., Aguilar-Manjarrez J., Cornish L., Dabbadie L., Desrochers A., Diffey S., Garrido Gamarro E., Geehan J., Hurtado A. (2021). Seaweeds and Microalgae: An Overview for Unlocking Their Potential in Global Aquaculture Development.

[42] Naik B., Mishra R., Kumar V., Mishra S., Gupta U., Rustagi S., Gupta A.K., Preet M.S., Bhatt S.C., Rizwanuddin S. (2024). Micro-Algae: Revolutionizing Food Production for a Healthy and Sustainable Future. J. Agric. Food Res..

[43] Su M., Bastiaens L., Verspreet J., Hayes M. (2023). Applications of Microalgae in Foods, Pharma and Feeds and Their Use as Fertilizers and Biostimulants: Legislation and Regulatory Aspects for Consideration. Foods.

[44] Peltomaa E., Johnson M.D., Taipale S.J. (2018). Marine Cryptophytes Are Great Sources of EPA and DHA. Mar. Drugs.

[45] Mansour M.P., Frampton D.M.F., Nichols P.D., Volkman J.K., Blackburn S.I. (2005). Lipid and Fatty Acid Yield of Nine Stationary-Phase Microalgae: Applications and Unusual C24-C28 Polyunsaturated Fatty Acids. J. Appl. Phycol..

[46] Odjadjare E.C., Mutanda T., Olaniran A.O. (2017). Potential Biotechnological Application of Microalgae: A Critical Review. Crit. Rev. Biotechnol..

[47] De Jesus Raposo M.F., De Morais R.M.S.C., De Morais A.M.M.B. (2013). Health Applications of Bioactive Compounds from Marine Microalgae. Life Sci..

[48] Varfolomeev S.D., Wasserman L.A. (2011). Microalgae as Source of Biofuel, Food, Fodder, and Medicines. Appl. Biochem. Microbiol..

[49] Guedes A.C., Amaro H.M., Malcata F.X. (2011). Microalgae as Sources of Carotenoids. Mar. Drugs.

[50] Gong M., Bassi A. (2016). Carotenoids from Microalgae: A Review of Recent Developments. Biotechnol. Adv..

[51] Mourelle M.L., Gómez C.P., Legido J.L. (2017). The Potential Use of Marine Microalgae and Cyanobacteria in Cosmetics and Thalassotherapy. Cosmetics.

[52] Paniagua-Miche J.D.J., Olmos-Soto J., Morales-Guerrero E. (2015). Microalgal Biotechnology: Biofuels and Bioproducts. Springer Handbook of Marine Biotechnology.

[53] Al-Jabri H., Das P., Khan S., Thaher M., Abdulquadir M. (2020). Treatment of Wastewaters by Microalgae and the Potential Applications of the Produced Biomass—A Review. Water.

[54] Desbois A.P. (2012). Potential Applications of Antimicrobial Fatty Acids in Medicine, Agriculture and Other Industries. Recent Pat. Antiinfect. Drug Discov..

[55] Maksimova I.V., Sidorova O.A. (1986). Light-Dependent Antibacterial Effect of Algae and Its Ecological Significance (a Review). Gidrobiol. Žurnal.

[56] Selivanova E.A., Ignatenko M.E., Nemtseva N. (2014). V Antagonistic Activity of Novel Green Microalgae Strain. Zhurnal Mikrobiol..

[57] Firoozabad M.S.M., Nasr M.M. (2022). Antimicrobial Activities of Microbial Essential Fatty Acid against Foodborne Pathogenic Bacteria. Iran. J. Microbiol..

[58] Cakmak Y.S., Kaya M., Asan-Ozusaglam M. (2014). Biochemical composition and bioactivity screening of various extracts from dunaliella salina, a green microalga. EXCLI J..

[59] Shannon E., Abu-Ghannam N. (2016). Antibacterial Derivatives of Marine Algae: An Overview of Pharmacological Mechanisms and Applications. Mar. Drugs.

[60] De Morais M.G., Vaz B.D.S., De Morais E.G., Costa J.A.V. (2015). Biologically Active Metabolites Synthesized by Microalgae. Biomed Res. Int..

[61] Maadane A., Merghoub N., El Mernissi N., Ainane T., Amzazi S., Wahby I., Bakri Y. (2017). Antimicrobial Activity of Marine Microalgae Isolated from Moroccan Coastlines. J. Microbiol. Biotechnol. Food Sci..

[62] Yoon B.K., Jackman J.A., Valle-González E.R., Cho N.J. (2018). Antibacterial Free Fatty Acids and Monoglycerides: Biological Activities, Experimental Testing, and Therapeutic Applications. Int. J. Mol. Sci..

[63] Chanda W., Joseph T.P., Guo X.F., Wang W.D., Liu M., Vuai M.S., Padhiar A.A., Zhong M.T. (2018). Effectiveness of Omega-3 Polyunsaturated Fatty Acids against Microbial Pathogens. J. Zhejiang Univ. Sci. B.

[64] Desbois A.P., Smith V.J. (2010). Antibacterial Free Fatty Acids: Activities, Mechanisms of Action and Biotechnological Potential. Appl. Microbiol. Biotechnol..

[65] Najdenski H.M., Gigova L.G., Iliev I.I., Pilarski P.S., Lukavský J., Tsvetkova I.V., Ninova M.S., Kussovski V.K. (2013). Antibacterial and Antifungal Activities of Selected Microalgae and Cyanobacteria. Int. J. Food Sci. Technol..

[66] Chaidir Z., Hillman P.F., Zainul R., Syafrizayanti (2016). Isolation and Identification of Freshwater Microalgae Potentially as Antibacterial from Talago Biru, Koto Baru, West Sumatera. Der Pharm. Lett..

[67] Alhusseiny S.M., El-Beshbishi S.N. (2020). Omega Polyunsaturated Fatty Acids and Parasitic Infections: An Overview. Acta Trop..

[68] Rao A.R., Reddy A.H., Aradhya S.M. (2010). Antibacterial Properties of Spirulina Platensis, Haematococcus Pluvialis, Botryococcus Braunii Micro Algal Extracts. Curr. Trends Biotechnol. Pharm..

[69] Rojas V., Rivas L., Cárdenas C., Guzmán F. (2020). Cyanobacteria and Eukaryotic Microalgae as Emerging Sources of Antibacterial Peptides. Molecules.

[70] Ishaq I.G., Matias-Peralta H.M., Basri H., Muhammad M.N. (2015). Antibacterial Activity of Freshwater Microalga *Scenedesmus* sp. on Foodborne Pathogens *Staphylococcus aureus* and *Salmonella* sp.. J. Sci. Technol..

[71] Bhagavathy S., Sumathi P., Jancy Sherene Bell I. (2011). Green Algae Chlorococcum Humicola-a New Source of Bioactive Compounds with Antimicrobial Activity. Asian Pac. J. Trop. Biomed..

[72] Bernal P., Llamas M.A. (2012). Promising Biotechnological Applications of Antibiofilm Exopolysaccharides. Microb. Biotechnol..

[73] Esko J.D., Sharon N. (2009). Microbial Lectins: Hemagglutinins, Adhesins, and Toxins. Essentials of Glycobiology.

[74] dos Santos Amorim R.N., Rodrigues J.A.G., Holanda M.L., Quinderé A.L.G., de Paula R.C.M., Melo V.M.M., Benevides N.M.B. (2012). Antimicrobial Effect of a Crude Sulfated Polysaccharide from the Red Seaweed Gracilaria Ornata. Brazilian Arch. Biol. Technol..

[75] De Jesus Raposo M.F., De Morais A.M.B., De Morais R.M.S.C. (2015). Marine Polysaccharides from Algae with Potential Biomedical Applications. Mar. Drugs.

[76] Rendueles O., Ghigo J.M. (2012). Multi-Species Biofilms: How to Avoid Unfriendly Neighbors. FEMS Microbiol. Rev..

[77] Nuhu A.A. (2013). Spirulina (Arthrospira): An Important Source of Nutritional and Medicinal Compounds. J. Mar. Biol..

[78] Alyasiri T., Al-Mayaly I., AL-Chalabi S. (2017). In Vitro and In Vivo Antibacterial Activity of Spirulina Platensis. Curr. Res. Microbiol. Biotechnol..

[79] Pratita A.T.K., Fathurohman M., Ruswanto R., Suhartati R., Khusnul (2019). Potential of Autotroph Microalgae (Spirulina Plantentis) as Antimicrobial Agent. J. Phys. Conf. Ser..

[80] Li A., Zhang L., Zhao Z.Y., Ma S.S., Wang M., Liu P.H. (2016). Prescreening, Identification and Harvesting of Microalgae with Antibacterial Activity. Biology.

[81] El-Sheekh M.M., El-Shafay S.M., El-Ballat E.M. (2016). In Vivo Evaluation of Antimicrobial Effect of Methanolic Extract of Chlorella Vulgaris on Impetigo and Some Dermatophytes. Egypt. J. Bot..

[82] Khadija A.T., Abd-Wahab R.A., Fadhil A.M. (2020). Effect of Ethanolic Extract of *Chlorella* sp on Entamoeba Histolytica Parasite in Vivo. Plant Arch..

[83] Herrero M., Ibáñez E., Cifuentes A., Reglero G., Santoyo S. (2006). Dunaliella Salina Microalga Pressurized Liquid Extracts as Potential Antimicrobials. J. Food Prot..

[84] González-Davis O., Ponce-Rivas E., Sánchez-Saavedra M.D.P., Muñoz-Márquez M.E., Gerwick W.H. (2012). Bioprospection of Microalgae and Cyanobacteria as Biocontrol Agents Against Vibrio Campbellii and Their Use in White Shrimp Litopenaeus Vannamei Culture. J. World Aquac. Soc..

[85] Kokou F., Makridis P., Kentouri M., Divanach P. (2012). Antibacterial Activity in Microalgae Cultures. Aquac. Res..

[86] Borowitzka M.A. (2018). Microalgae in Medicine and Human Health: A Historical Perspective. Microalgae in Health and Disease Prevention.

[87] Ayswaria R., Vijayan J., Nathan V.K. (2023). Antimicrobial Peptides Derived from Microalgae for Combating Antibiotic Resistance: Current Status and Prospects. Cell Biochem. Funct..

[88] Martelli F., Cirlini M., Lazzi C., Neviani E., Bernini V. (2020). Edible Seaweeds and Spirulina Extracts for Food Application: In Vitro and In Situ Evaluation of Antimicrobial Activity towards Foodborne Pathogenic Bacteria. Foods.

[89] Sun Y., Chang R., Li Q., Li B. (2015). Isolation and Characterization of an Antibacterial Peptide from Protein Hydrolysates of Spirulina Platensis. Eur. Food Res. Technol..

[90] Ganapathy K., Ramasamy R., Dhinakarasamy I. (2018). Polyhydroxybutyrate Production from Marine Source and Its Application. Int. J. Biol. Macromol..

[91] Kuntzler S.G., de Almeida A.C.A., Costa J.A.V., Morais M.G. (2018). de Polyhydroxybutyrate and Phenolic Compounds Microalgae Electrospun Nanofibers: A Novel Nanomaterial with Antibacterial Activity. Int. J. Biol. Macromol..

[92] Kroumov A.D., Módenes A.N., Trigueros D.E.G., Espinoza-Quiñones F.R., Borba C.E., Scheufele F.B., Hinterholz C.L. (2016). A Systems Approach for CO2 Fixation from Flue Gas by Microalgae—Theory Review. Process Biochem..

[93] Scheufele F.B., Hinterholz C.L., Zaharieva M.M., Najdenski H.M., Módenes A.N., Trigueros D.E.G., Borba C.E., Espinoza-Quiñones F.R., Kroumov A.D. (2019). Complex Mathematical Analysis of Photobioreactor System. Eng. Life Sci..

[94] Gonçalves V.D., Fagundes-Klen M.R., Goes Trigueros D.E., Kroumov A.D., Módenes A.N. (2019). Statistical and Optimization Strategies to Carotenoids Production by Tetradesmus Acuminatus (LC192133.1) Cultivated in Photobioreactors. Biochem. Eng. J..

[95] Gonçalves V.D., Fagundes-Klen M.R., Trigueros D.E.G., Schuelter A.R., Kroumov A.D., Módenes A.N. (2019). Combination of Light Emitting Diodes (LEDs) for Photostimulation of Carotenoids and Chlorophylls Synthesis in *Tetradesmus* sp.. Algal Res..

[96] Lemoine Y., Schoefs B. (2010). Secondary Ketocarotenoid Astaxanthin Biosynthesis in Algae: A Multifunctional Response to Stress. Photosynth. Res..

[97] Kobayashi M. (2003). Astaxanthin Biosynthesis Enhanced by Reactive Oxygen Species in the Grlen Alga Haematococcus Pluvialis. Biotechnol. Bioprocess Eng..

[98] Del Campo J.A., García-González M., Guerrero M.G. (2007). Outdoor Cultivation of Microalgae for Carotenoid Production: Current State and Perspectives. Appl. Microbiol. Biotechnol..

[99] Shariati M., Hadi M.R., Carpi A. (2011). Microalgal Biotechnology and Bioenergy in Dunaliella. Progress in Molecular and Environmental Bioengineering—From Analysis and Modeling to Technology Applications.

[100] Lamers P.P., Janssen M., De Vos R.C.H., Bino R.J., Wijffels R.H. (2012). Carotenoid and Fatty Acid Metabolism in Nitrogen-Starved Dunaliella Salina, a Unicellular Green Microalga. J. Biotechnol..

[101] Ben-Amotz A. (1987). Effect of Irradiance and Nutrient Deficiency on the Chemical Composition of Dunaliella Bardawil Ben-Amotz and Avron (Volvocales, Chlorophyta). J. Plant Physiol..

[102] Phadwal K., Singh P.K. (2003). Effect of Nutrient Depletion on β-Carotene and Glycerol Accumulation in Two Strains of *Dunaliella* sp.. Bioresour. Technol..

[103] Ben-Amotz A., Seckbach J. (1999). Dunaliella β-Carotene From Science to Commerce. Enigmatic Microorganisms and Life in Extreme Environments.

[104] Ben-Amotz A., Tornabene T.G., Thomas W.H. (1985). Chemical Profile of Selected Species of Microalgae with Emphasis on Lipids1. J. Phycol..

[105] Hosseini Tafreshi A., Shariati M. (2009). Dunaliella Biotechnology: Methods and Applications. J. Appl. Microbiol..

[106] Ben-Amotz A., Richmond A. (2003). Industrial Production of Microalgal Cell-Mass and Secondary Products—Major Industrial Species: Dunaliella. Handbook of Microalgal Culture.

[107] Coesel S.N., Baumgartner A.C., Teles L.M., Ramos A.A., Henriques N.M., Cancela L., Varela J.C.S. (2008). Nutrient Limitation Is the Main Regulatory Factor for Carotenoid Accumulation and for Psy and Pds Steady State Transcript Levels in Dunaliella Salina (Chlorophyta) Exposed to High Light and Salt Stress. Mar. Biotechnol..

[108] Mishra A., Mandoli A., Jha B. (2008). Physiological Characterization and Stress-Induced Metabolic Responses of Dunaliella Salina Isolated from Salt Pan. J. Ind. Microbiol. Biotechnol..

[109] Ben-Amotz A. (1996). Effect of Low Temperature on the Stereoisomer Composition of β-carotene in the Halotolerant Alga Dunaliella Bardawil (Chlorophyta)1. J. Phycol..

[110] Gómez P.I., González M.A. (2005). The Effect of Temperature and Irradiance on the Growth and Carotenogenic Capacity of Seven Strains of Dunaliella Salina (Chlorophyta) Cultivated under Laboratory Conditions. Biol. Res..

[111] Ben-Amotz A., Avron M. (1983). On the Factors Which Determine Massive Beta-Carotene Accumulation in the Halotolerant Alga Dunaliella Bardawil. Plant Physiol..

[112] Lamers P.P., Van De Laak C.C.W., Kaasenbrood P.S., Lorier J., Janssen M., De Vos R.C.H., Bino R.J., Wijffels R.H. (2010). Carotenoid and Fatty Acid Metabolism in Light-Stressed Dunaliella Salina. Biotechnol. Bioeng..

[113] Fu W., Guomundsson Ó., Paglia G., Herjólfsson G., Andrésson Ó.S., Palsson B.O., Brynjólfsson S. (2013). Enhancement of Carotenoid Biosynthesis in the Green Microalga Dunaliella Salina with Light-Emitting Diodes and Adaptive Laboratory Evolution. Appl. Microbiol. Biotechnol..

[114] Li Z., Ma X., Li A., Zhang C. (2012). A Novel Potential Source of β-Carotene: Eustigmatos Cf. Polyphem (Eustigmatophyceae) and Pilot β-Carotene Production in Bubble Column and Flat Panel Photobioreactors. Bioresour. Technol..

[115] Li Z., Sun M., Li Q., Li A., Zhang C. (2012). Profiling of Carotenoids in Six Microalgae (Eustigmatophyceae) and Assessment of Their β-Carotene Productions in Bubble Column Photobioreactor. Biotechnol. Lett..

[116] Sohani E., Pajoum Shariati F., Pajoum Shariati S.R. (2023). Assessment of Various Colored Lights on the Growth Pattern and Secondary Metabolites Synthesis in Spirulina Platensis. Prep. Biochem. Biotechnol..

[117] Chentir I., Doumandji A., Ammar J., Zili F., Jridi M., Markou G., Ben Ouada H. (2018). Induced Change in Arthrospira sp (Spirulina) Intracellular and Extracellular Metabolites Using Multifactor Stress Combination Approach. J. Appl. Phycol..

[118] Caroprese M., Ciliberti M.G., Albenzio M. (2015). Immunological Activity of Marine Microalgae Extracts. Marine Algae Extracts: Processes, Products, and Applications.

[119] Riccio G., Lauritano C. (2020). Microalgae with Immunomodulatory Activities. Mar. Drugs.

[120] Hirahashi T., Matsumoto M., Hazeki K., Saeki Y., Ui M., Seya T. (2002). Activation of the Human Innate Immune System by Spirulina: Augmentation of Interferon Production and NK Cytotoxicity by Oral Administration of Hot Water Extract of Spirulina Platensis. Int. Immunopharmacol..

[121] Al-Ghanayem A.A. (2023). Effect of Methanol Extracts of Arthrospira Platensis on Survival and Increased Disease Resistance in Litopenaeus Vannamei against Vibriosis. J. Pure Appl. Microbiol..

[122] Marques A., Thanh T.H., Sorgeloos P., Bossier P. (2006). Use of Microalgae and Bacteria to Enhance Protection of Gnotobiotic Artemia against Different Pathogens. Aquaculture.

[123] Watanuki H., Ota K., Tassakka A.C.M.A.R., Kato T., Sakai M. (2006). Immunostimulant Effects of Dietary Spirulina Platensis on Carp, Cyprinus Carpio. Aquaculture.

[124] Duncan P., Klesius P. (2011). Effects of Feeding Spirulina on Specific and Nonspecific Immune Responses of Channel Catfish. J. Aquat. Anim. Health.

[125] Tayag C.M., Lin Y.C., Li C.C., Liou C.H., Chen J.C. (2010). Administration of the Hot-Water Extract of Spirulina Platensis Enhanced the Immune Response of White Shrimp Litopenaeus Vannamei and Its Resistance against Vibrio Alginolyticus. Fish Shellfish Immunol..

[126] Chen Y.Y., Chen J.C., Tayag C.M., Li H.F., Putra D.F., Kuo Y.H., Bai J.C., Chang Y.H. (2016). Spirulina Elicits the Activation of Innate Immunity and Increases Resistance against Vibrio Alginolyticus in Shrimp. Fish Shellfish Immunol..

[127] Vántus V., Bónai A., Zsolnai A., Bosco A.D., Szendrő Z., Tornyos G., Bodnár Z., Morsy W.A., Pósa R., Toldi M. (2023). Single and Combined Effect of Dietary Thyme (Thymus Vulgaris) and Spirulina (Arthrospira Platensis) on Bacterial Community in the Caecum and Caecal Fermentation of Rabbits. ACTA Agric. Slov..

[128] Dalle Zotte A., Celia C., Szendro Z. (2016). Herbs and Spices Inclusion as Feedstuff or Additive in Growing Rabbit Diets and as Additive in Rabbit Meat: A Review. Livest. Sci..

[129] Chellapandian H., Sivakamavalli J., Anand A.V., Balasubramanian B., Chellapandian H., Sivakamavalli J., Anand A.V., Balasubramanian B. (2021). Challenges in Controlling Vibriosis in Shrimp Farms. Infections and Sepsis Development.

[130] Chrisolite B., Thiyagarajan S., Alavandi S.V., Abhilash E.C., Kalaimani N., Vijayan K.K., Santiago T.C. (2008). Distribution of Luminescent Vibrio Harveyi and Their Bacteriophages in a Commercial Shrimp Hatchery in South India. Aquaculture.

[131] Khimmakthong U., Sukkarun P. (2017). The Spread of Vibrio Parahaemolyticus in Tissues of the Pacific White Shrimp Litopenaeus Vannamei Analyzed by PCR and Histopathology. Microb. Pathog..

[132] Zorriehzahra M.J. (2015). Early Mortality Syndrome (EMS) as New Emerging Threat in Shrimp Industry. Adv. Anim. Vet. Sci..

[133] Hu Y., Lei D., Wu D., Xia J., Zhou W., Cui C. (2022). Residual β-Lactam Antibiotics and Ecotoxicity to Vibrio Fischeri, Daphnia Magna of Pharmaceutical Wastewater in the Treatment Process. J. Hazard. Mater..

[134] Abou-Zeid E.H.F., Bedair A.M., Figueroa F.L., Salas J.A., Gheda S., Abd El-Zaher E.H.F., Abou-Zeid A.M., Bedair N.A., Pereira L. (2023). Potential Activity of Arthrospira Platensis as Antioxidant, Cytotoxic and Antifungal against Some Skin Diseases: Topical Cream Application. Mar. Drugs.

[135] Li W., Wang S., Zhong D., Du Z., Zhou M. (2021). A Bioactive Living Hydrogel: Photosynthetic Bacteria Mediated Hypoxia Elimination and Bacteria-Killing to Promote Infected Wound Healing. Adv. Ther..

[136] Hu H., Zhong D., Li W., Lin X., He J., Sun Y., Wu Y., Shi M., Chen X., Xu F. (2022). Microalgae-Based Bioactive Hydrogel Loaded with Quorum Sensing Inhibitor Promotes Infected Wound Healing. Nano Today.

[137] Loke M.F., Lui S.Y., Ng B.L., Gong M., Ho B. (2007). Antiadhesive Property of Microalgal Polysaccharide Extract on the Binding of Helicobacter Pylori to Gastric Mucin. FEMS Immunol. Med. Microbiol..

[138] Pina-Pérez M.C., Úbeda-Manzanaro M., Beyrer M., Martínez A., Rodrigo D. (2022). In Vivo Assessment of Cold Atmospheric Pressure Plasma Technology on the Bioactivity of Spirulina. Front. Microbiol..

[139] Rathi Bhuvaneswari G., Shukla S.P., Makesh M., Thirumalaiselvan S., Arun Sudhagar S., Kothari D.C., Singh A. (2013). Antibacterial Activity of Spirulina (Arthospira Platensis Geitler) against Bacterial Pathogens in Aquaculture. Isr. J. Aquac.-Bamidgeh.

[140] Ahmad M.T., Shariff M., Yusoff F.M., Goh Y.M., Banerjee S. (2020). Applications of Microalga Chlorella Vulgaris in Aquaculture. Rev. Aquac..

[141] de Souza M.M., Prietto L., Ribeiro A.C., de Souza T.D., Badiale-Furlong E. (2011). Assessment of the Antifungal Activity of Spirulina Platensis Phenolic Extract against Aspergillus Flavus. Cienc. Agrotecnologia.

[142] Catarina Guedes A., Barbosa C.R., Amaro H.M., Pereira C.I., Xavier Malcata F. (2011). Microalgal and Cyanobacterial Cell Extracts for Use as Natural Antibacterial Additives against Food Pathogens. Int. J. Food Sci. Technol..

[143] Kumar V. (2011). Antibacterial Activity of Crude Extracts of Spirulina Platensis and Its Structural Elucidation of Bioactive Compound. J. Med. Plants Res..

[144] El-Sheekh M.M., Daboo S., Swelim M.A., Mohamed S. (2014). Production and Characterization of Antimicrobial Active Substance from Spirulina Platensis. Iran. J. Microbiol..

[145] Oguzkan S.B., Guroy B.K., Tonus S.S., Guroy D., Kılıc H.I. (2018). The Bioactive Component and DNA Protective Capability of Cultured Spirulina in Turkey (Marmara Region). Genet. Aquat. Org..

[146] Manigandan M., Kolanjinathan K. (2017). Antibacterial Activity of Various Solvent Extracts of Spirulina Platensis against Human Pathogens. Innovare J. Heal. Sci..

[147] Sudha S.S., Karthic R., Rengaramanujam J. (2011). Athulya Antimicrobial Activity of Spirulina Platensis and. South As. J. Biol. Sci..

[148] Alghanmi H.A., Omran A.S. (2020). Antibacterial Activity of Ethanol Extracts of Two Algae Species against Some Pathogenic Bacteria Isolated from Hospital Patients. Eur. Asian J. Biosci..

[149] Sadeghi S., Jalili H., Ranaei Siadat S.O., Sedighi M. (2018). Anticancer and Antibacterial Properties in Peptide Fractions from Hydrolyzed Spirulina Protein. J. Agric. Sci. Technol..

[150] Elshouny W.A.E.F., El-Sheekh M.M., Sabae S.Z., Khalil M.A., Badr H.M. (2017). Antimicrobial Activity of Spirulina Platensis against Aquatic Bacterial Isolates. J. Microbiol. Biotechnol. Food Sci..

[151] da Silva Júnior J.N., de Aguiar E.M., Mota R.A., Bezerra R.P., Porto A.L.F., Herculano P.N., de Araújo Viana Marques D. (2019). Antimicrobial Activity of Photosynthetic Microorganisms Biomass Extract against Bacterial Isolates Causing Mastitis. J. Dairy Vet. Sci..

[152] Hamouda I.A., Doumandji A. (2017). Comparative Phytochemical Analysis and in Vitro Antimicrobial Activities of the Cyanobacterium Spirulina Platensis and the Green Alga Chlorella Pyrenoidosa: Potential Application of Bioactive Components as an Alternative to Infectious Diseases. Bull. l’Institut Sci..

[153] Ahsan S., Arefin M.S., Munshi J.L., Begum M.N., Maliha M., Rahman S., Bhowmik A., Kabir M.S. (2016). In Vitro Antibacterial Activity of Spirulina Platensis Extracts against Clinical Isolates of Salmonella Enterica Serovars Typhi and Paratyphi (SUBP03). Stamford J. Microbiol..

[154] Abo-State M.A.M., Shanab S.M.M., Ali H.E.A., Abdullah M.A. (2015). Screening of Antimicrobial Activity of Selected Egyptian Cyanobacterial Species. J. Ecol. Heal. Environ. An Int. J..

[155] Mudimu O., Rybalka N., Bauersachs T., Born J., Friedl T., Schulz R. (2014). Biotechnological Screening of Microalgal and Cyanobacterial Strains for Biogas Production and Antibacterial and Antifungal Effects. Metabolites.

[156] Shaieb F.A., Issa A.A.-S., Meragaa A. (2014). Antimicrobial Activity of Crude Extracts of Cyanobacteria Nostoc Commune and Spirulina Platensis. Arch. Biomed. Sci..

[157] El-Sheekh M.M., El-Shafaay S.M., Abou-Shady A.M., El-Ballat E.M. (2014). Antibacterial Activities of Different Extracts of Some Fresh and Marine Algae. Egypt. J. Exp. Biol..

[158] Sivakumar J., Santhanam P. (2011). Antipathogenic Activity of Spirulina Powder. Recent Res. Sci. Technol..

[159] Sarada D.V.L., Kumar C.S., Rengasamy R. (2011). Purified C-Phycocyanin from Spirulina Platensis (Nordstedt) Geitler: A Novel and Potent Agent against Drug Resistant Bacteria. World J. Microbiol. Biotechnol..

[160] Pradhan J., Das B.K., Sahu S., Marhual N.P., Swain A.K., Mishra B.K., Eknath A.E. (2011). Traditional Antibacterial Activity of Freshwater Microalga Spirulina Platensis to Aquatic Pathogens. Aquac. Res..

[161] Challouf R., Trabelsi L., Ben Dhieb R., El Abed O., Yahia A., Ghozzi K., Ben Ammar J., Omran H., Ben Ouada H. (2011). Evaluation of Cytotoxicity and Biological Activities in Extracellular Polysaccharides Released by Cyanobacterium Arthrospira Platensis. Braz. Arch. Biol. Technol..

[162] Uyisenga J., Nzayino P., Seneza R., Hishamunda L., Uwantege K., Gasana N., Bajyana E. (2010). In Vitro Study of Antibacterial and Antifungal Activity of Spirulina Platensis. Int. J. Ecol. Dev..

[163] Prakash S., Sasikala S.L., Aldous V.H.J. (2010). Isolation and Identification of MDR-Mycobacterium Tuberculosis and Screening of Partially Characterised Antimycobacterial Compounds from Chosen Marine Micro Algae. Asian Pac. J. Trop. Med..

[164] Pradhan J., Das B.K. (2010). Antibacterial Properties of Selected Freshwater Microalgae against Pathogenic Bacteria. Ind. J. Fish..

[165] Devi K.N., Dhayanithi N.B., Kumar T.T.A., Balasundaram C., Harikrishnan R. (2016). In Vitro and in Vivo Efficacy of Partially Purified Herbal Extracts against Bacterial Fish Pathogens. Aquaculture.

[166] Velichkova K., Sirakov I., Rusenova N., Beev G., Denev S., Valcheva N., Dinev T. (2018). In Vitro Antimicrobial Activity on Lemna Minuta, Chlorella Vulgaris and Spirulina sp Extracts. Fresenius Environ. Bull..

[167] Dang V.T., Li Y., Speck P., Benkendorff K. (2011). Effects of Micro and Macroalgal Diet Supplementations on Growth and Immunity of Greenlip Abalone, Haliotis Laevigata. Aquaculture.

[168] Medina-Jaritz N.B., Pérez-Solis D., S.L.RuilobadeLeon F., Olvera-Ramírez R., Mendez-Vilas A. (2011). Antimicrobial Activity of Aqueous and Methanolic Extracts from Arthrospira Maxima. Science Against Microbial Pathogens: Communicating Current Research and Technological Advances.

[169] Abd El-Baky H.H., El-Baroty G.S. (2012). Characterization and Bioactivity of Phycocyanin Isolated from Spirulina Maxima Grown under Salt Stress. Food Funct..

[170] Ambrico A., Trupo M., Magarelli R., Balducchi R., Ferraro A., Hristoforou E., Marino T., Musmarra D., Casella P., Molino A. (2020). Effectiveness of Dunaliella Salina Extracts against Bacillus Subtilis and Bacterial Plant Pathogens. Pathogens.

[171] El-Baz F.K., Salama A., Ali S.I., El-Hashemy H.A. (2023). Dunaliella Salina Chitosan Nanoparticles as a Promising Wound Healing Vehicles: In-Vitro and in-Vivo Study. OpenNano.

[172] Jang S.H., Lim J.W., Kim H. (2009). β-Carotene Inhibits Helicobacter Pylori-Induced Expression of Inducible Nitrix Oxide Synthase and Cyclooxygenase-2 in Human Gastric Epithelial Cells. J Physiol Pharmacol..

[173] Ahmad S.A., Yong J.F.S., Syamsumir D.F., Zin N.A.M., Radzi S.A.M., Kassim M.N.I., Muzamel M.A., Yusof M.R., Segaran T.C. (2015). The Potential of Carotenoids from Marine Tropical Microalgae in the Healing Process of Gastritis. J. Sustain. Sci. Manag..

[174] Iglesias M.J., Soengas R., Probert I., Guilloud E., Gourvil P., Mehiri M., López Y., Cepas V., Gutiérrez-del-Río I., Redondo-Blanco S. (2019). NMR Characterization and Evaluation of Antibacterial and Antiobiofilm Activity of Organic Extracts from Stationary Phase Batch Cultures of Five Marine Microalgae (*Dunaliella* sp, *D. Salina*, *Chaetoceros Calcitrans*, *C. Gracilis* and *Tisochrysis Lutea*). Phytochemistry.

[175] Krishnakumar S., Dooslin V., Bai M., Rajan A.R.A. (2013). Evaluation of Bioactive Metabolites from Halophilic Microalgae Dunaliella Salina by GC-MS Analysis. Int. J. Pharm. Pharm. Sci..

[176] Chakraborty K. (2023). Recent Advances in Marine Biotechnology. Frontiers in Aquaculture Biotechnology.

[177] Montalvão S., Demirel Z., Devi P., Lombardi V., Hongisto V., Perälä M., Hattara J., Imamoglu E., Tilvi S.S., Turan G. (2016). Large-Scale Bioprospecting of Cyanobacteria, Micro- and Macroalgae from the Aegean Sea. N. Biotechnol..

[178] Pane G., Cacciola G., Giacco E., Mariottini G.L., Coppo E. (2015). Assessment of the Antimicrobial Activity of Algae Extracts on Bacteria Responsible of External Otitis. Mar. Drugs.

[179] Jafari S., Mobasher M.A., Najafipour S., Ghasemi Y., Mohkam M., Ebrahimi M.A., Mobasher N. (2018). Antibacterial Potential of Chlorella Vulgaris and Dunaliella Salina Extracts against Streptococcus Mutans. Jundishapur J. Nat. Pharm. Prod..

[180] Widowati I., Zainuri M., Kusumaningrum H.P., Maesaroh Y., Hardivillier Y., Leignel V., Bourgougnon N., Mouget J.-L. (2018). Identification of Agents Causing Vibriosis in Litopenaeus Vannamei Shrimp Culture in Kendal, Central Java, Indonesia and Application of Microalgae Dunaliella Salina and Tetraselmis Chui as Bio-Control Agents against Vibriosis. AACL Bioflux.

[181] del Pilar Sánchez-Saavedra M., Licea-Navarro A., Bernáldez-Sarabia J. (2010). Evaluation of the Antibacterial Activity of Different Species of Phytoplankton. Rev. Biol. Mar. Oceanogr..

[182] Eloff J.N. (2004). Quantification the Bioactivity of Plant Extracts during Screening and Bioassay Guided Fractionation. Phytomedicine.

[183] Aligiannis N., Kalpoutzakis E., Mitaku S., Chinou I.B. (2001). Composition and Antimicrobial Activity of the Essential Oils of Two Origanum Species. J. Agric. Food Chem..

[184] Greenwood D. (1985). The Alteration of Microbial Growth Curves by Antibiotics. Rapid Methods and Automation in Microbiology and Immunology.

[185] Kurniawati I., Adam A., Zamzami I., Maftuch (2016). Antibacterial Effect of Gracilaria Verrucosa Bioactive on Fish Pathogenic Bacteria. Egypt. J. Aquat. Res..

[186] Genovese G., Faggio C., Gugliandolo C., Torre A., Spanò A., Morabito M., Maugeri T.L. (2012). In Vitro Evaluation of Antibacterial Activity of Asparagopsis Taxiformis from the Straits of Messina against Pathogens Relevant in Aquaculture. Mar. Environ. Res..

